# Maternal–Fetal Transfer and Toxicokinetics of 2,2′,5,5′-Tetrachlorobiphenyl, [^14^C]-PCB52, Following Intratracheal Administration

**DOI:** 10.1021/acs.chemrestox.5c00265

**Published:** 2025-11-03

**Authors:** Yau Adamu, Andrea Adamcakova-Dodd, Xuefang Jing, Dustin May, Peter S. Thorne

**Affiliations:** †Human Toxicology Program, ‡Department of Occupational and Environmental Health, §State Hygienic Laboratory, 4083 The University of Iowa, Iowa City, Iowa 52242, United States

## Abstract

Despite increased recognition of the adverse impacts of PCB exposure on human health, comprehensive risk assessments, particularly regarding inhalation exposure and effects on the developing fetus, are lacking. Out of all PCB congeners, lower-chlorinated PCBs have been more prevalent in indoor and outdoor atmospheres. Thus, we investigated *in vivo* toxicokinetics and placental transfer of radiolabeled [^14^C]-PCB52 (0.157 mg/kg administered intratracheally) in Sprague–Dawley rats at gestational day 11 ± 1. Following dosing, 99.4 ± 0.5% of the administered dose was distributed to the systemic circulation. Radioactivity disappeared biexponentially following lung exposure, with 41.1% of the dose retained after 96 h. PCB52 was rapidly distributed to the maternal serum, lung, heart, and liver, with subsequent accumulation in the ovaries, brain, white and brown adipose, muscle, and mammary glands. The time to reach a maximum concentration in the maternal serum was 0.21 h, with an apparent terminal elimination half-life of 40.7 h. The peak concentration of [^14^C]-PCB52 and its metabolites in the placenta, fetus, and amniotic fluid was achieved 1.7 h after exposure, with a fetal half-life of 34.8 h. The maternal serum level was significantly correlated with levels in amniotic fluid, placenta, fetus, and the maternal brain. However, PCB52 exposure in the placenta, fetus, and amniotic fluid was limited with their respective maternal serum exposure ratio values of 0.5, 0.27, and 0.05. These results demonstrate for the first time a comprehensive whole-body disposition of PCB52 in dams and fetuses after lung exposure during gestation. PCB52 and its metabolites accumulate predominantly in the ovaries, brain, and mammary glands. The apparent half-life of PCB52 in developing fetuses and placenta is comparable to that of maternal serum. This study provides novel quantitative foundations for the development and evaluation of physiologically based toxicokinetic modeling to inform the exposure and risk assessment for public health decisions.

## Graphical abstract

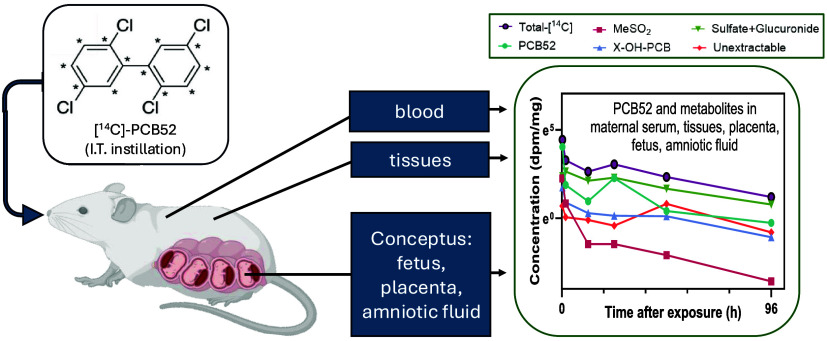

## 1 Introduction

Polychlorinated biphenyls (PCBs) are a group of persistent organic pollutants comprising 209 congeners with endocrine-disruptive toxicity. Despite the ban on commercial production, restrictions, and documented toxicity of PCBs, inhalation exposure to semivolatile PCBs that ubiquitously exist in the urban atmosphere and indoor environment remains prevalent.^1–5^ Those PCBs with four or fewer chlorine atoms (lower-chlorinated, LC-PCBs), especially PCB52, dominate the PCBs found in indoor and outdoor air in school and residential settings.^6–9^ LC-PCBs have been linked to several health effects, including reproductive, genotoxic, and neurodevelopmental toxicity characterized by impaired learning, behavior, and intellectual development in children.^10–14^

Pregnant individuals and their developing fetuses are the most vulnerable and sensitive populations to adverse effects from environmental exposures.^15,16^ The placenta serves as the interface between mother and fetus and takes part in the control and passage of nutrients, xenobiotics, and antigens.^16,17^ Many PCB congeners can cross the placenta to reach the fetus; however, significantly higher exposure levels have been reported through breastfeeding than *in utero*.^18–21^ *In utero* exposure to LC-PCBs presents unique and significant risks to the fetus. Prenatal exposure to PCBs may affect fetal development through several mechanisms. These include direct accumulation in the fetal tissues and subsequent adverse effects,^22–25^ altering the function of the placenta, or indirectly by causing uterine muscles to contract and are associated with shortened gestation and reduced birth weight.^13,26,27^ These pathways can reduce the supply of oxygen and nutrients to the fetus and may contribute to adverse outcomes such as preterm labor and delivery. Although breastfeeding can result in substantial PCB exposure,^28,29^ prenatal exposure remains a critical window of vulnerability due to the sensitivity of developing organ systems and the potential for long-term effects.^29^ Several human biomonitoring studies demonstrated that the levels of LC-PCBs and total PCBs in maternal serum are positively correlated with those in umbilical cord serum, suggesting transplacental transfer as a critical pathway for prenatal exposure of the fetus.^20,21,30^ However, several of those biomonitoring studies reported undetected levels of LC-PCBs in umbilical cord blood samples, particularly PCB52, in the same cohort with elevated levels of higher-chlorinated PCB congeners (HC-PCBs) in analyzed samples of human cord blood.^31–34^

Animal experiments and human biomonitoring studies have demonstrated that *in utero* exposure to LC-PCBs is associated with neurodevelopmental toxicity, characterized by impaired learning, behavioral, or intellectual development in children.^10,11,13,14,35,36^ However, the available findings from the PCB placental transfer investigation were limited to higher-chlorinated PCBs (HC-PCBs) and *ex vivo* human biomonitoring studies, which used analysis of the umbilical cord blood samples as a surrogate of fetal exposure levels.^37,38^ The toxicological profiles of HC-PCBs and LC-PCBs differ significantly,^39^ and caution is warranted when extrapolating data from HC-PCBs to LC-PCBs. Historically, PCB risk assessments have been based on HC-PCB data due to limited LC-PCB studies.^39–41^ This underscores the importance of expanding research on LC-PCBs. Moreover, congener-specific toxicity varies not only with number of chlorines but also with exposure routes, substitution patterns, and structural features such as planarity and receptor binding.^42–46^ These differences underscore the need for congener-specific assessments,^47,48^ specifically for *in vivo* studies elucidating the uptake, absorption, distribution, metabolism, excretion, and transplacental transfer of individual LC-PCB congeners, particularly from studies that model inhalational exposure during early pregnancy. This study provides much-needed toxicokinetic (TK) data on PCB52, elucidating maternal plasma TK parameters, placental transfer, and fetal exposure to enable linking airborne PCB exposure to body burdens of specific inhaled PCB congeners and inform risk estimation for PCBs.

## 2 Materials and Methods

### 2.1 Chemicals

PCB52, uniformly labeled with carbon-14 on the biphenyl ring with a specific activity of 60.0 mCi/mmol and radiochemical purity exceeding 99%, was custom-produced by Vitrax, Placentia, CA. The reference unlabeled PCBs and their corresponding metabolites used in this study were synthesized and provided by the Iowa Superfund Research Program (iSRP) Synthesis Core. The detailed synthetic procedures and characterizations of those reference unlabeled PCBs and their corresponding metabolites have been documented.^49–52^ Multipurpose Ultima Gold liquid scintillation cocktails (catalog no. 6013329) and SOLVABLE solution (catalog No. 140-22271) were purchased from Revvity, Inc. (Waltham, MA, USA). All reagents and solvents used for extractions and analysis were of pesticide-grade quality and were procured from Sigma-Aldrich (St. Loius, MO, USA).

### 2.2 Preparation of [^14^C]-Labeled PCB52 Exposure Solution

A stock of [^14^C]-PCB52 solution was prepared by dissolving 0.5 mCi of [^14^C]-PCB52 in 100 μL of hexane and was stored at −20 °C. Fresh 1 mL quantities of the dosing solution were prepared by adding 10 μL of hexane to 10 μL of the stock solution, followed by the addition of sterile saline in five aliquots. Sonication was performed between each addition until an emulsion formed. The final [^14^C]-PCB52 dosing solution consisted of 2% hexane and 0.1% Tween 80 in saline. During intratracheal dosing, an equivalent portion of the administered solution was analyzed for radioactivity using a liquid scintillation counter (Beckman LS 6500, Fullerton, CA) to ensure consistent dosing across the study animals.

### 2.3 Animal Study and Exposure

Female Sprague–Dawley rats (8 weeks old) weighing 237–269.7 g (mean = 247.5 g) (Envigo RMS, LLC, Indianapolis, IN) were used for this study. All animal experiments were performed following the protocols evaluated and approved by the University of Iowa Institutional Animal Care and Use Committee (approval number 3091097). After arrival, the animals were acclimated for 7 days. The animals were housed in a light and temperature-controlled environment with *ad libitum* access to food and water during quarantine and throughout the study. Timed pregnancy was achieved by harem mating.^53,54^ Briefly, one male and two female rats were placed together in the evening and checked for vaginal plug the following day.^55^ If vaginal plug was present, the male was removed from the breeding cage, and the gestation day 0 (GD 0), 0-day post coitum was assigned. Pregnancies were confirmed by ultrasound scan at GD 9.^55^ The dams were exposed at GD 11 (±1 day) to capture the period of early organogenesis and hormonal function, which falls within week 3 of human embryonic development.^16,17,56–58^ On the day of the experiment, pregnant rats were intratracheally administered an average of 40 μg (range, 35–45 μg) of [^14^C]-PCB52 in 100 μL of exposure solution. Although the dose used in this study exceeded the tolerable daily intake (TDI) of PCBs for healthy individuals, which is 1.6 μg per day for an individual weighing 80 kg,^59^ the use of higher acute doses has been established in toxicological studies intended for risk assessment.^60^ The intratracheal instillation method was chosen for administering [^14^C]-PCB52 to represent inhalation exposures, capture exhaled concentration, and efficiently utilize the limited availability of the cost-intensive radiolabeled PCB52.^61,62^ Although inhalation exposure would better represent real-world exposures to airborne LC-PCBs, it would not allow us to accurately measure the amount of PCB52 exhaled into the air after exposure and is experimentally infeasible. Compared to other routes of exposure, the intratracheal route exhibited a higher absorption efficiency, leading to higher bioavailability.^63–65^ We used tissue samples from dams exposed to unlabeled cold PCB52 to establish the background levels for the radioactivity calculations in dams exposed to [^14^C]-PCB52. All intratracheal instillations were done under light anesthesia, achieved using a 4% isoflurane concentration via a Fortec vaporizer (Cyprane, Keighley, UK). The animals recovered from anesthesia within 1 min. Subsequently, the animals were housed within a cylindrical glass chamber measuring 2 L in volume, equipped with glass drainage mechanisms to facilitate urine collection and a metallic screen to segregate urine from feces (as in Figure S1). Air was drawn from the chamber at 6.0 L/min through a cartridge containing the XAD-2 polymeric resin to capture any exhaled [^14^C]-PCB52. For periods longer than 24 h postexposure, exposed dams were placed in a metabolic cage, which was fitted with a cartridge containing XAD-2 to capture exhaled [^14^C]-PCB52 as in other end points.

Maternal blood, urine, and feces samples were collected immediately before and at 0.21, 1.67, 12, 24, 48, and 96 h after lung dosing. The exposed dams were euthanized using 4% isoflurane, followed by cervical dislocation and exsanguination via a cardiac puncture. Euthanasia was consistently performed during the light cycle. Dams’ fur was wiped and analyzed for radioactivity. Using the retro-orbital blood collection method, 0.5–1.5 mL (1% of an exposed pregnant rat’s body weight) of blood sample was collected from each of the dams until the individual dams were necropsied for whole-body tissue harvesting. At the end of the exposure period, the chamber’s interior walls were rinsed separately with water and acetone/hexane (25 – 45 mL) and wiped to measure residues. From the collected whole blood samples, 100 μL were aliquoted into three vials each, and the remaining blood was allowed to clot at room temperature before being centrifuged to yield serum. The placentas, developing fetuses, and whole-body tissue samples were harvested immediately before exposure and at 0.21, 1.67, 12, 24, and 96 h postexposure. Three unexposed pregnant rats (control group) were handled in the same manner as the exposed animals in the experiments, except for the absence of [^14^C]-PCB52 instillation. The collected tissue samples were placed in a scintillation vial containing 1 mL of SOLVABLE cocktail (catalog no. 6NE9100, Revvity, Inc., Waltham, MA) and kept at 37 °C overnight for complete solubilization. The solubilized tissue samples underwent a 2 h heating and gentle shaking phase at 50 °C. For the feces and digestive matter, 1 mL of isopropyl alcohol was added. For the samples that appeared darker in coloration, a controlled volume of 30% hydrogen peroxide (ranging from 100 to 300 μL) was added to remove the potential color-based interference during the analysis. Subsequently, 10 mL of scintillation cocktail (Ultima Gold, catalog no.: 6013329, Revvity) was added to each sample, and the quantification of radioactivity was performed using a liquid scintillation counter. The extracts from the acetone/hexane and water rinses were analyzed to quantify the residual radioactivity in the glass chamber. To ensure accurate quantification, we used quench correction curves established through the utilization of a [^14^C]-toluene standard to ensure activity counting efficiency and accurate quantification (Revvity, Inc., Waltham, MA, USA). Amniotic fluid samples were obtained by drawing the fluid using a 23-G needle. The placenta and fetuses were removed by dissection, weighed, and then homogenized in SOLVABLE. Harvested serum, whole blood, amniotic fluid, and urine were kept at −20 °C until analysis, while harvested placenta, fetuses, maternal tissue samples, and digestive matter were kept at −80 °C until analysis. All radioactive materials were used in accordance with the University of Iowa’s radiation safety program requirements.

### 2.4 Analysis of [^14^C]-PCB52 Total Radioactivity

Aliquots of serum, whole blood, fetus, placenta, urine, digestive matter, feces, and cage rinse-wash samples from rats were taken for direct liquid scintillation counting. The total [^14^C]-labeled radioactivity in the samples was quantified with a Revvity Tri-Carb 4910TR (Serial Number SGLO28180804). The gross count rates were corrected for background, detection efficiency, and quenching (using the tSIE as an indicator of quench) to convert counts per minute (cpm) to disintegrations per minute (dpm). The extraction recovery of PCB52 and its metabolites ranged from 60 to 99.3% (mean = 80.3 ± 5.3%).

### 2.5 Metabolite Extraction and Analysis

A complex mixture of confirmed and putative metabolites of PCBs has been reported in the literature.^66^ To quantify the metabolites of PCB52 in maternal serum, the developing fetus, liver, brain, lung, and adipose tissue in this study, we employed a series of liquid–liquid extractions with solvents of increasing polarity to separate phenolic, methyl sulfones, and glucuronate/sulfated metabolites.^4,62^ Recovery experiments used available standards including parent PCB52, 4-OH-PCB52, 3-MeSO_2_-PCB52, and 4-MeSO_2_-PCB52 metabolites in biological samples. The levels of the parent compound PCB52 and its extractable metabolites in the samples were determined using a validated liquid scintillation counting method coupled with gas chromatography-electron capture detection (GC-ECD), following our previous protocol.^4,62^ Individual extractable metabolite was separated using a series of liquid–liquid extractions, employing a graded array of solvents with incrementing polarity to facilitate the partitioning of the analytes according to their inherent lipophilic characteristics (Figure S4). Subsequently, individual fractions were classified into respective categories, including the parent PCB moiety, hydroxylated PCBs (OH-PCBs), methyl sulfonyl PCBs (MeSO_2_–PCBs), conjugated metabolites, and unextractable components.

Tissue samples (200–400 mg wet weight) were prepared for analysis through a two-step homogenization using 2 mL of hexane/acetone solvent (50% vol/vol). The resultant solution was centrifuged, and then the pellet underwent two successive extractions with 1.5 mL of dichloromethane (DCM), chloroform/methanol (50:50 vol/vol), methanol/water (50:50 vol/vol), and water, respectively. The supernatant from the DCM extraction contained hydroxylated metabolites, whereas the top fractions from the methanol/water extraction had conjugated PCB metabolites. The resultant supernatant from the hexane/acetone extraction was rinsed with Milli-Q water, then extracted with a 0.5 M potassium hydroxide (KOH) solution in 50% ethanol, and triple rinsed with 400 μL of hexane. The hydrophobic fraction was further purified by rinsing with 1 M hydrochloric acid (400 μL). Simultaneously, a plausible MeSO_2_–PCBs fraction was extracted with 500 μL of anhydrous DMSO, followed by triple hexane rinses (3 × 400 μL). The residual hydrophobic phase was quantified as native PCB52. The quantification of cold PCB52, OH-PCBs (as MeO-PCB derivatives), and MeSO2-PCBs standards in spiked samples was initially performed on a gas chromatograph (Agilent 6890N) equipped with a 63Ni μ-ECD detector and SLB-5MS capillary column (60 m × 0.25 mm I.D., 0.25 μm film thickness; Supelco, St Louis, MO). Subsequently, individual separated fractionation from [^14^C]-PCB52-exposed tissue samples was transferred to a prepared scintillation vial, followed by the addition of 10 mL of cocktail solution and analysis by scintillation counter. Any quantifiable radioactivity in the residual pellet was treated as unextractable compounds.

### 2.6 Data Analysis

The sample size of the study was not based on hypothesis testing. Instead, it was based on previous studies of toxicokinetics and mass balance.^4,67^ Findings from those studies demonstrated that 3 rats per time point for the plasma toxicokinetic study were sufficient to obtain adequate absorption, distribution, metabolism, and excretion information, while minimizing the number of required animals for the study and ensuring efficient use of very expensive [^14^C]-PCB52.

We performed descriptive statistical analysis of the total [^14^C]-PCB52 concentrations of individual tissue types. The concentration versus time data of multiple organs were plotted on semilog scales using Prism 10.2.0 (GraphPad Software, Boston, MA 02110). The total radioactivity of each organ was calculated as the sum of radioactivity found in each sample multiplied by the organ weight. For the feces and digestive matter, radioactivity was taken as the product of the sample concentration and total wet weight divided by total dry weight. Total weights for adipose tissue, skin, muscle, bone, and blood were determined based on documented percentage body weights of 6, 16, 44, 7, and 6%, respectively.^68^ The exhaled amount of radioactivity was composed of the amount in the XAD cell cartridge, the chamber rinses and wipes, and the rat wipes. The degree of pulmonary absorption was calculated by subtracting exhalation and fecal excretion data from 100% of the nominal dose administered to each dam and calculating the mean percentage and apparent absorption efficiency.^69^ Toxicokinetic parameters were estimated using the Phoenix Pharmacokinetic and Pharmacodynamic (PK/PD) Platform Version 8.4.^70,71^ Briefly, a noncompartmental analysis (NCA) was conducted on the plasma concentration–time data resulting from the administration of [^14^C]-PCB52 via the intratracheal route. The essential parameters of interest were derived from these data. Specifically, the maximal concentration (*C*_max_) of PCB52 in plasma and the corresponding time point (*T*_max_) at which it was attained were determined. The elimination rate constant (Ke) was estimated by fitting the terminal log–linear segment of the plasma concentration–time curve, employing a least-squares approach. The area under the plasma concentration–time curve within the 0 to 24 h interval (AUC_0–24_) was computed utilizing the linear trapezoidal rule. Correspondingly, the area under the curve spanning from time zero to infinity (AUC_0‑∞_) was extrapolated to infinity using the following equation: AUC_0–24_ + C_24_/Ke, with C_24_ signifying the plasma concentration measured at the 24 h postexposure time point. The dose-normalized total area under the curve (AUC_0‑∞/dose_) was computed to account for potential dose variations. The terminal elimination half-life (λ_*z*_) was estimated by using the estimated apparent terminal rate constant. The partition coefficient (*P*_tissue/blood_) of PCB52 in different tissues following intratracheal administration was calculated using the area method^72,73^ by taking the ratios of the AUC_0‑∞_tissue_ in each tissue to the AUC_0‑∞_blood_ in blood as reported previously.^69,74,75^

## 3 Results and Discussion

### 3.1 Pulmonary Uptake and Tissue Disposition of [^14^C]-PCB52 over Time

The average lung uptake of [^14^C]-PCB52 following intratracheal administration was 99.40 ± 0.51%. The exhaled proportion of the administered dose was 1.33 ± 0.51% by the end of the study period (96 h postexposure). Following a near-complete lung uptake, the activity rapidly distributed to the systemic circulation. The tissue distribution was evaluated based on the concentration distribution of [^14^C]-PCB52 per mg of wet tissue (Figure 1A1,A2) and percent distribution (Supporting Information Table S1).

**Figure 1:**
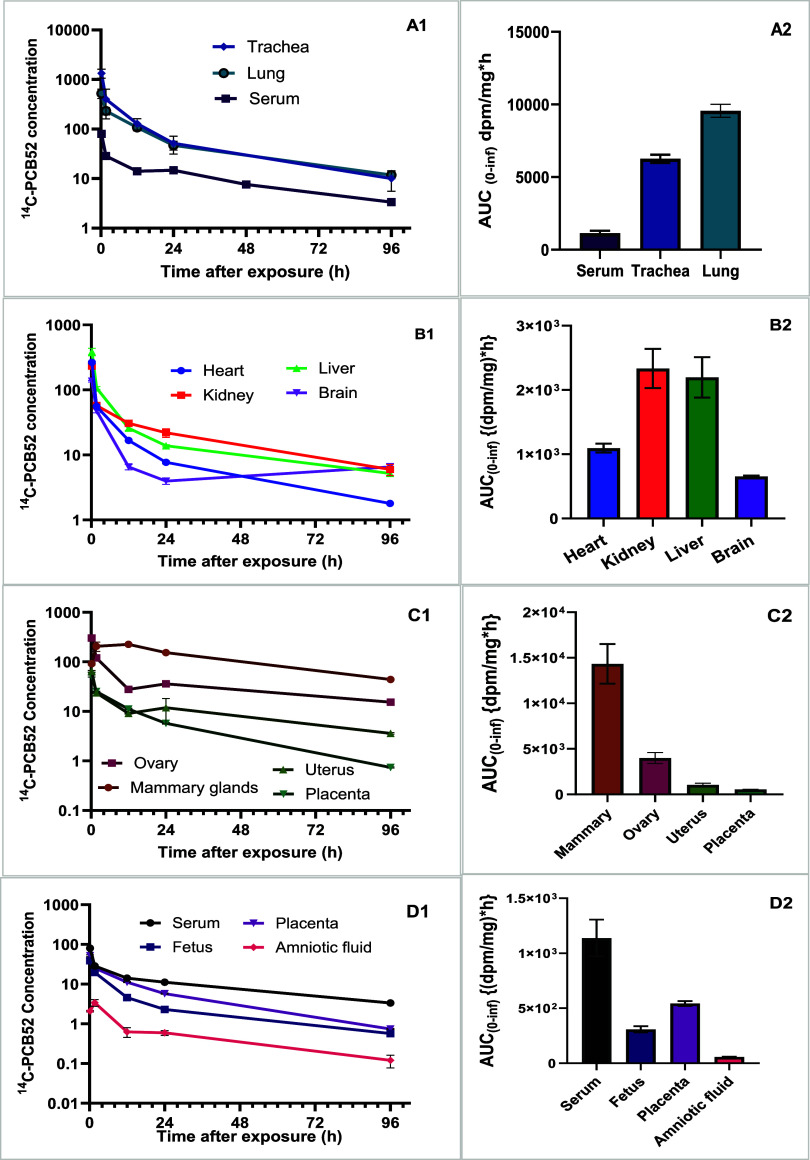
Time course of [^14^C]­PCB52 concentration (dpm/mg wet tissue) change in exposed dams showing lung uptake in trachea, lung, and serum (*n* = 4) (A1 and A2), distribution into the liver, kidney, heart, and brain compartments (B1 and B2), distribution to reproductive tissues (C1 and C2), and exposure levels in the fetus, placenta, and amniotic fluid (D1 and D2).

Following rapid distribution throughout the body, including placentas, developing fetuses, and amniotic fluids (Figures 1A1–D2 and 2E1–G2), the highest *C*_max_ in dpm per mg of wet tissue was recorded in the lung at 0.21 h postexposure (Figure 1A1 and Table S2). The pituitary gland compartment took the longest time to achieve *C*_max_ (*T*_max_ = 96 h), followed by the adipose tissue, mammary gland, and gastrointestinal tract content (Table S2). PCB52 concentration in the brain declined rapidly from 0.21 h postexposure and started accumulating from 24 h postexposure, whereas a continued decline in concentration was observed in the heart, liver, and kidneys (Figure 1B1).

**Figure 2:**
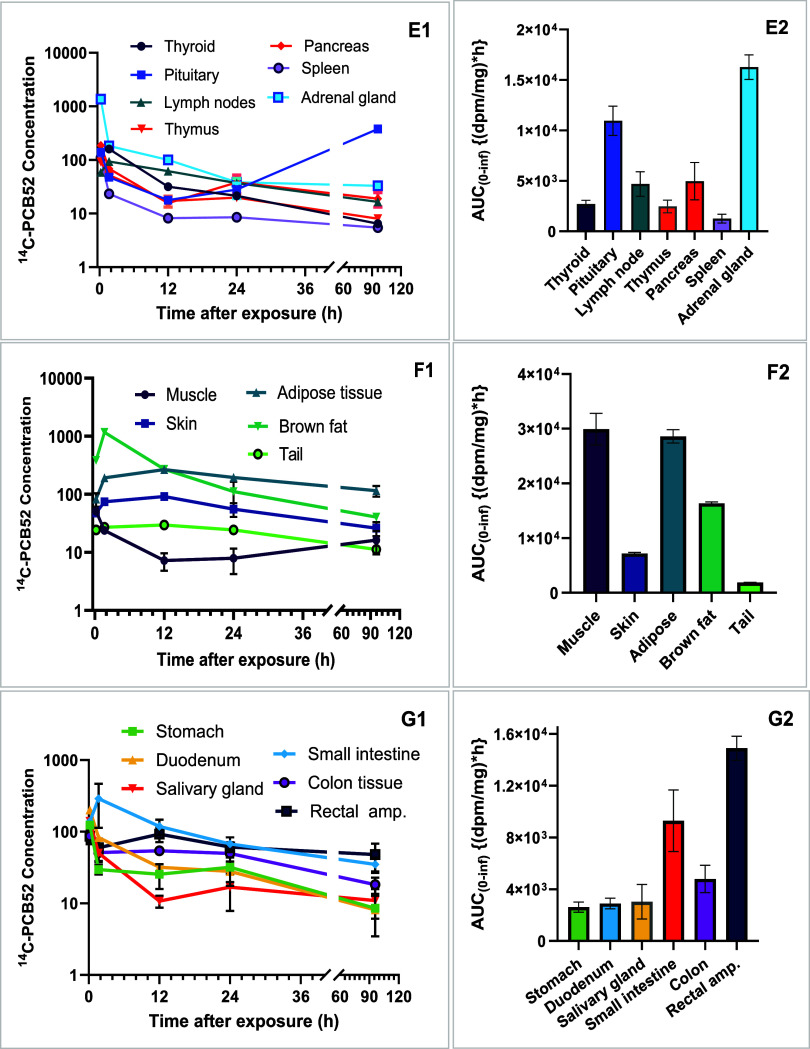
Arithmetic mean concentration (dpm/mg wet tissue) versus time profiles and total radioactivity of [^14^C]-PCB52 showing disposition in the pituitary and adrenal glands, thyroid, thymus, spleen, and pancreas with corresponding exposure levels (E1 and E2), biphasic distribution in the brown fat and tail, little to nonchanging levels in the adipose, muscle, and skin (F1) with corresponding exposure profiles (F2). (G1 and G2) show a multiphasic distribution with corresponding exposure levels in the gastrointestinal tissues.

The time-course disposition of [^14^C]-PCB52 in the reproductive system demonstrated a rapid distribution to the mammary glands at 0.21 h after exposure, followed by a steep decline from 1.7 h postexposure (Figure 1C1 and Table S2). The remaining reproductive organs demonstrated a continuous decline in concentration of total [^14^C]-PCB52, with the highest exposure level recorded in the ovaries, followed by the uterus and the placenta (Figure 1C2).

The time-course concentration and exposure levels in the developing fetuses versus maternal serum and placenta are shown in Figure 1D1,D2. At 96 h (4 days) postexposure; the concentrations in fetuses were comparable to those in placentas (Figure 1D1). We observed a sharp increase in the activity of PCB52 in developing fetuses from 0.2 to 1.7 h, followed by a steady concentration between 12 and 24 h and continued decline up to 96 h postexposure (Figure 1D1,D2). We observed comparable exposure levels in fetuses and placentas. The concentration versus time profiles and exposure levels of PCB52 in the endocrine system, including adrenal glands, thyroid, thymus, spleen, pancreas, and pituitary glands, are displayed in Figure 2E1,E2, and show biphasic elimination profiles in all endocrine organs. There was little to no elimination from the spleen, pancreas, and adrenal gland compartments from 24 h postexposure to the end of our observation period. We observed accumulation in the pituitary gland from 12 h postexposure up to 96 h postexposure. The concentration and exposure levels of PCB52 in adipose tissue differed between white and brown adiposes (Figure 2F1,F2). The time to achieve *C*_max_ was shorter in the brown compared to the white adipose (Table 1 and Figure 2F1). We observed little to no elimination of total activity from the adipose, muscle, and skin compartments. The distribution of PCB52 in the GI tract follows multiphasic patterns with maximum concentrations achieved within 0.21 h in the stomach, duodenum, and salivary gland compartments (Figure 2G1).

**Table 1:** Mean Toxicokinetic Parameters of PCB52 Obtained by NonCompartmental Analysis after IntraTracheal Administration of [^14^C]-PCB52

parameters	unit	maternal serum	placenta	fetus	amniotic fluid
*C*_max_	dpm/mL	0.080	0.025	0.020	0.003
*T*_max_, h	h	0.21	1.67	1.67	1.67
AUC_0–96h_	h*dpm/mL	0.97	0.52	0.28	0.052
AUC_0‑∞_	h*dpm/mL	1.14	0.54	0.31	0.056
AUC_% extrap	%	14.2	4.1	10.7	7.8
λ_*z*_	h	34.6	21.1	34.8	26.4
CL/F_obs	mL/h	0.018	NA	NA	NA
Vz/F_obs	mL	0.26	NA	NA	NA

In the gastrointestinal tissue, the levels of PCB52 demonstrate an absorption phase up to 1.67 h after dosing in the small intestine. We observed a slight decline in activity at the rectal ampulla and colon tissues, with early [^14^C] activity decline in the stomach, small intestine, and colon tissues. The rectal ampulla region of the GI tract experienced the highest levels of accumulation (Figure 2G2).

The time-course concentration changes in exposed dams show a multiphasic distribution, along with exposure levels of [^14^C] in the digestive matter contents and elimination via feces (Figure 3A1,A2). The highest level was measured in feces, which was the major route of elimination. During the initial phase, the concentration was higher in the food content in the duodenum, followed by food in the stomach (Figure 3A1).

**Figure 3:**
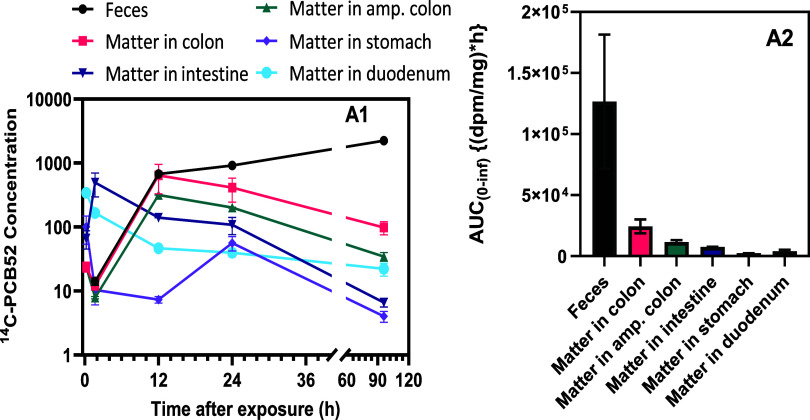
Time course of concentration (dpm/mg wet tissue) changes in exposed dams showing a multiphasic distribution and exposure levels of [^14^C] in the digestive matter contents and elimination via feces after pulmonary exposure to [^14^C]-PCB52 (Figure 3A1 and A2).

The percent distribution describes the distribution of radioactivity into various tissue compartments regardless of the mass proportion of each tissue compartment to the body mass, while the concentration distribution takes into account the mass proportion of each tissue compartment (Table S1). At the earliest time point (0.21 h postexposure), the highest percentage was recovered from the muscle, followed by liver, adipose tissue, skin, blood, and lung compartments (muscle > liver > adipose > skin > blood), whereas the concentrations per mg of wet tissues were in this order; trachea > bronchi > lung > liver > brown adipose (Table S1). At 12 h postadministration, the percent activity distribution patterns changed, with the highest percent activity recovered from the white adipose tissue, followed by the skin, muscle, and digestive matter in the colon and ampullar colon compartments. The percentage distribution of total activity 24 h postexposure remained highest in the white adipose tissue, followed by skin and muscle tissues. Similarly, the concentration per mg of wet tissue after 24 h demonstrated the following order: matter in the colon > white adipose tissue> matter in the ampullar colon > mammary gland > brown adipose tissue> matter in the intestine > stomach and small intestine tissue. By the end of the study (96 h postexposure), the highest percentage was found in the skin, followed by adipose tissue. In terms of concentration per mg of wet tissue, the highest concentration was found in the matter content in the colon, followed by matter in the ampullar colon, adipose tissue, mammary gland, and matter in the intestinal compartments.

### 3.2 Toxicokinetic Parameters of [^14^C]-PCB52

The toxicokinetic parameters of PCB52 are presented in Table 1. The analysis of maternal serum data demonstrated an apparent λ_*z*_ of 1.7 days with a clearance-to-bioavailability ratio (Cl/F) of 0.018 mL/h. The *T*_max_ and time to achieve the maximum plasma concentration (*C*_max_) are presented in Table 1. The areas under the serum concentration–time curve from time zero to the last observation time (AUC_0–96 h_) and from zero to infinity were 0.97 and 1.14 h*dpm/mL, respectively. Unlike the whole-body serum, the *T*_max_ for the brain, fetuses, amniotic fluids, placentas, uteruses, and ovaries was 1.67 h. The apparent λ_*z*_ in the placenta was observed to be 21.1 h. *T*_max_ in the placenta was 0.020 dpm/mg, with respective AUC_0–96 h_ and AUC_0‑∞_ values of 0.52 and 0.54 dpm/mL.

The λ_*z*_ was relatively shorter in the developing fetuses than in the maternal serum, with a *C*_max_ of 0.020 dpm/mL achieved within 1.67 h after the dose. The Cl/F and Vz/F were not determinable in the fetuses because the actual dose reaching the developing fetus was not available.

AUC_0‑∞_, area under the concentration–time curve from zero up to ∞, with extrapolation of the terminal phase, was calculated with the equation AUC_0‑∞_ = AUC_0–24_ + C_last_/Ke using known observed concentration and estimated terminal elimination half-life values. Cl/F, apparent total serum clearance of ^14^C-PCB52 after a single dose estimated from CL/F = Dose/AUC_0‑∞_; Vz/F, apparent volume of distribution during the terminal phase for nonsteady-state after extravascular administration from Vz/F = Dose__ex_/(AUC_0‑∞_*Ke).

Compared with the fetus, a lower *C*_max_ of 0.003 dpm/mL was achieved in the amniotic fluid within the same *T*_max_ of 1.67 h. A relatively shorter λ_*z*_ of 26.4 h was observed in the amniotic fluid than in the maternal serum, with the amniotic fluid exposure level expressed in terms of AUC_0–96 h_ and AUC_0‑∞_ (Table 1). The exposure levels in the ovary and mammary gland expressed by AUC_0‑∞_ were significantly different from the maternal serum (Figure 4). *T*_max_ values in the lung, heart, kidney, liver, muscle, pancreas, spleen, thymus, esophagus, and gastrointestinal tissues were achieved by 0.21 h (Table S2).

**Figure 4:**
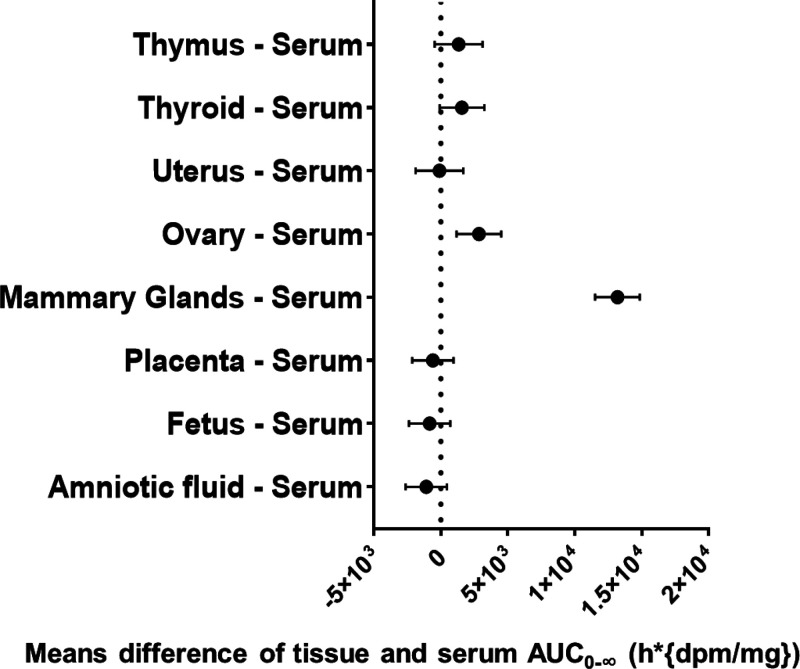
Forest plot showing the difference in AUC_o‑∞_ (h*dpm/mL) means between maternal serum and fetus, placenta, and other tissues, with corresponding 95% confidence intervals (CI).

### 3.3 Ratios of Toxicokinetic Parameters as a Measure of Placental Transfer

The partitioning property of [^14^C]-PCB52 from maternal serum to placenta, developing fetus, and amniotic fluid is shown in Table 2. Briefly, the observed experimental blood-to-plasma ratio of [^14^C]-PCB52 was 0.53. The tissue partitioning demonstrated efficiency ranges from the least partitioning in the amniotic fluid to the maternal serum and fetus to the maternal serum compartments to the most partitioning in the maternal adipose tissue to the serum compartment (Table S2). To assess the roles of the placental barrier in the transfer of [^14^C]-PCB52 from dams to developing fetuses after acute lung dosing, we calculated the ratios of *C*_max_, AUC_0–96_, and extrapolated AUC_0–96_ parameters of placenta to maternal serum, fetus to maternal serum, amniotic fluid to maternal serum, fetus to placenta, amniotic fluid to placenta, and fetus to amniotic fluid (Table 2). The amniotic fluid-to-maternal plasma ratios of *C*_max_, AUC_0–96_, and AUC_0‑∞_ were the lowest, followed by the amniotic fluid-to-placenta and then amniotic fluid-to-fetus ratios, which contrasted with the *C*_max_ ratio of the amniotic fluid-to-fetus ratio, suggesting a greater partitioning of [^14^C]-PCB52 into the fetus from amniotic fluid. This finding is supported by the AUC_0–96_ and AUC_0‑∞_ ratios, which provide an estimate of the total exposure to [^14^C]-PCB52 over the entire duration of the study. The *C*_max_ ratio (and AUC_0–96_ /AUC_0‑∞_ ratios) of placenta-to-maternal serum and fetus-to-placenta were less than 1, except the amniotic fluid to fetus ratio, suggesting the placenta does act as to partially shield the fetus. The PCB52 levels in maternal serum are significantly correlated with the concentration in the placenta, fetus, amniotic fluid, pituitary gland, thymus, and brain (Table 2 and Figure S2).

**Table 2:** Ratios of Toxicokinetic Parameters as a Measure of Placental Transfer^a^

tissue ratios	*C*_max_ ratio	AUC_0–96h_ ratio	AUC_0‑∞_ ratio	coefficient (R)
uterus/MS	0.296	0.888	0.917	0.625
ovary/MS	1.512	3.043	0.251	–0.224
placenta/MS	0.316	0.536	0.477	0.635*
AF/MS	0.043	0.053	0.050	0.224*
fetus/MS	0.135	0.100	0.104	0.381*
pituitary/MS	4.741	11.289	ND	0.784*
thymus/MS	1.152	1.903	2.169	–0.399*
thyroid/MS	2.005	2.435	2.387	0.985
brain/MS	0.598	0.672	ND	0.161*
mammary gland/MS	2.822	12.52	12.59	–0.399*
fetus/placenta	0.778	0.538	0.569	–0.356*
fetus/AF	5.767	5.405	5.472	0.980**
fetus/ovary	0.163	0.094	0.078	–0.107
fetus/thymus	0.314	0.152	0.125	0.143*
fetus/pituitary	0.052	0.026	ND	0.923**
uterus/ovary	0.196	0.292	0.262	–0.228
pituitary/thymus	4.114	5.933	ND	–0.058
fetus/mammary gland	11.47	43.42	46.45	–0.099
fetus/thyroid	8.150	8.445	8.805	0.992**
AF/brain	0.071	0.079	ND	0.982*
brain/thyroid	0.298	0.276	ND	0.985*

a MS; maternal serum, AF; amniotic fluid, R; Pearson correlation, * *P* < 0.05, ** *P* < 0.01.

### 3.4 Metabolism and Metabolites

PCB52 and its metabolites were quantified in maternal serum, liver, placenta, fetuses, brain, lung, adipose tissue, and urine over a 96 h period following intratracheal administration. The disposition of phenol, methyl sulfones, glucuronides, and sulfate metabolites in the maternal serum, liver, placenta, developing fetus, brain, lung, adipose tissue, and urine was quantified in exposed dams over this period (Figure 5A–H). The metabolism of PCB52 occurs rapidly, with detection in the liver, maternal serum, placenta, and lung tissues as early as 0.21 h postexposure. The distribution and metabolic profiles of PCB52 and its metabolites varied significantly across maternal and fetal compartments, reflecting the distinct physiological roles and metabolic capacities of each tissue. Within the first 24 h postexposure, parent PCB52 remained the dominant compound in the placenta and fetus, but not in the maternal serum (Figure 5A), where the predominant compounds were the sulfate and glucuronide metabolites. After 24 h, PCB52 and sulfate and glucuronide metabolites remained dominant in the maternal serum and placenta, whereas the hydroxylated metabolite was the major metabolite in the fetus.

**Figure 5:**
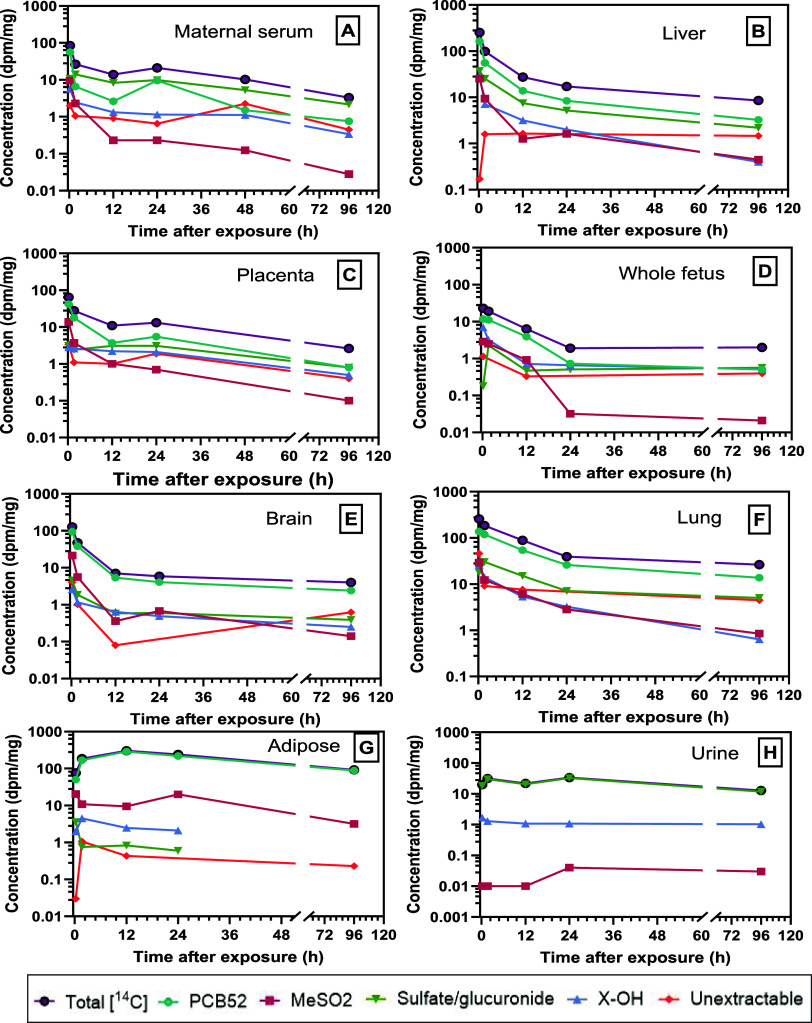
Time-course change representing parent PCB52 and its metabolites in different tissues following a single dose of [14C]-PCB52 via lung instillation. Each graph represents moiety distribution in maternal serum (A), liver (B), placenta (C), whole developing fetus (D), brain (E), lung (F), adipose tissue (G), and urine (H).

In maternal serum (Figure 5A), PCB52 declined rapidly from 69.4% at 0.21 h to 6.8% at 96 h, accompanied by a substantial rise in sulfate/glucuronide conjugates and OH-PCBs, indicating efficient systemic metabolism and clearance. The liver retained higher levels of unmetabolized PCB52 over time (64.0 to 37.3%), demonstrating elevated levels of OH-PCBs and conjugates in the liver compared to other compartments (Figure 5B), which confirms its central role in both phase I and phase II metabolism. The placenta showed intermediate retention of PCB52 (67.8 to 28.6%) and increasing levels of OH-PCBs and conjugates, reflecting both metabolic activity and its role in maternal–fetal exchange (Figure 5C).

In fetal tissues (Figure 5D), PCB52 declined from 54.1 to 27.7%, contrary to the increased levels of OH-PCBs and sulfate/glucuronide conjugates, indicating both transplacental transfer and concentration dilution due to fetal growth during early pregnancy. The brain retained high levels of unmetabolized PCB52 (74.4 to 60.2%) with limited formation of OH-PCBs and conjugates, consistent with the blood–brain barrier’s restrictive nature and the brain’s low metabolic capacity (Figure 5E). However, the nondeclining levels suggest potential for bioaccumulation or binding of reactive metabolites. The lung (Figure 5F), as the site of administration, exhibited moderate retention of PCB52, accompanied by increasing levels of OH-PCBs and conjugate metabolites, along with notable unextractable [^14^C]-PCB52 activity (up to 25.5%), suggesting local metabolism and potential tissue binding. In adipose tissue (Figure 5G), PCB52 was highly retained (66.0 to 96.8%) with minimal metabolic transformation, consistent with its lipophilic nature and low enzymatic activity, suggesting a long-term storage role. Finally, the urine contained no detectable parent compound but was dominated by sulfate and glucuronide conjugates (92.4 to 96.7%), confirming metabolic excretion as the primary elimination route for phase II metabolites (Figure 5H).

Collectively, these profiles illustrate a coordinated system of distribution, metabolism, and clearance, with the liver and serum facilitating transformation and transport, the placenta and fetus reflecting exposure and vulnerability, the brain and adipose tissue representing retention and storage, and urine serving as the terminal route for metabolite elimination. At 96 h postexposure, 62.8, 47.5, and 53.3% of the parent PCB52 was converted to sulfate/glucuronide and methyl sulfone (phase II metabolites) in maternal serum, placenta, and fetus, respectively. Contrary to the metabolism of other congeners,^76,77^ methyl sulfonyl PCB52 was not the dominant metabolite. Instead, radioactivity primarily stems from phase II metabolites circulating in the maternal blood, placenta, and developing fetus. PCBs with fewer chlorine atoms (low molecular weight, LC-PCBs) and the presence of two adjacent unsubstituted carbon atoms undergo faster metabolism to phase II metabolites, compared with LC-PCBs without vicinal carbons on the biphenyl structure.

## 4 Discussion

This study presents pioneering results on the *in vivo* disposition of PCB52 in maternal tissues and developing fetuses following lung exposure. Our results inform our prior research that demonstrated inhalation as a critical route of exposure for LC-PCBs such as PCB52, due to their higher prevalence in residential indoor and school air samples compared to their higher-chlorinated counterparts.^6–8^ This study’s findings align with existing evidence suggesting that inhaled PCBs bypass intestinal and hepatic first-pass metabolism, allowing for direct distribution via the bloodstream to target tissues. We demonstrated that PCB52 absorption efficiency following intratracheal exposure is much higher than the absorption rates previously reported from studies of oral exposures to Aroclor and PCB congeners, where serum levels did not peak until 2 to 12 h postexposure.^78^ Following lung exposure, PCB52 was rapidly absorbed and distributed to the maternal tissues, placenta, fetus, and amniotic fluid, with peak concentrations reached within 1.67 h, while the maximum concentration in maternal blood was achieved quickly, at 0.21 h. The ability of PCB52 to cross the placental barrier and reach the fetus highlights the risk for developmental toxicity. Remarkably, the concentration of PCB52 in the brain exhibited accumulative behavior 24 h postexposure. This pattern contrasts with the continued decline in the heart, liver, and kidney.

The accumulation of PCB52 in the brain and pituitary glands provides a plausible explanation for the documented neurodevelopmental disorders and endocrine disruption associated with PCB exposures.^77,79^ The brain is a crucial target site for PCB neurotoxicity. The nonmonotonic PK profile observed in the brain and pituitary at 96 h postexposure compared to 24 h suggests tissue-specific retention mechanisms that differ from systemic clearance patterns. While serum levels of PCB52 steadily decline over time, the delayed increase in radioactivity in these two tissues demonstrates a combination of physicochemical and biological factors that may govern accumulation. One plausible explanation is delayed partitioning of PCB52 or its metabolites into lipid-rich compartments of the brain and pituitary. Both tissues are characterized by high lipid content and specialized barriers, which may slow the initial uptake but facilitate prolonged retention of PCB52. Existing literature demonstrates that exposure to either individual PCB congeners or an Aroclor mixture of PCBs can alter blood–brain barrier integrity by disruption of tight junction function, resulting in increased permeability, potentially facilitating metabolite entry into the brain.^80–83^ PCB52, a lipophilic compound, may gradually diffuse into these compartments, resulting in a time-dependent increase in the tissue concentration despite declining systemic levels. Second, the increase in unextractable components observed in the brain at 96 h may reflect the formation of covalently bound or tightly associated PCB52 species. These could include adducts with proteins, lipids, or nucleic acids that are not readily extractable but retain radiolabel, thereby contributing to the total radioactivity signal. These covalent binding interactions and formation have been reported in a few studies,^84–87^ and the covalent adducts formation was more pronounced in noncoplanar PCBs like PCB52.^88^ The parallel rise in unextractable fractions and total radioactivity supports the hypothesis that bioactivation and binding events may play a role in tissue-specific retention. Notably, while the brain shows limited presence of extractable OH-PCBs and conjugated metabolites, consistent with its low metabolic capacity and the restrictive nature of the barrier, this does not preclude the possibility that circulating metabolites formed in peripheral tissues may cross into the brain and accumulate. These metabolites, particularly hydroxylated or methyl sulfones (MeSO2), and conjugated species,^89–91^ may possess structural features that enhance receptor binding or disrupt endocrine and neural signaling pathways. Thus, even in the absence of local metabolism, the brain may be exposed to neurotoxic PCB52 derivatives, explaining the apparent contradiction between the low brain metabolism and the proposed role of metabolites in neurotoxicity. Altogether, our findings suggest that the observed accumulation in the brain and pituitary gland may be driven by a combination of slow tissue uptake, limited clearance, and formation of persistent or bioactive species. This has important implications for understanding the long-term effects of PCB52 exposure during pregnancy, particularly in the context of neurodevelopmental and endocrine outcomes.^24,40,79,81,92^ Future studies using tissue-specific metabolite profiling and covalent binding assays will be critical to elucidate the precise mechanisms underlying this accumulation.

The position and number of chlorine substitutions on the biphenyl rings of each PCB congener influence the potential mechanistic interactions between individual PCB congeners and their target molecules, leading to adverse effects. Dioxin-like PCBs exhibit a coplanar structure like that of 2,3,7,8-TCDD and interact with the aryl hydrocarbon receptor (AhR) pathway to produce adverse effects.^44,93–97^ In contrast, other PCBs, without a coplanar structure due to chlorine substitution at the ortho position, have a different toxicological profile, involving several distinct mechanisms of toxicity. PCB52 belongs to the group of PCB congeners with two or more chlorine substitutions at the ortho position, suggesting a lack of interaction with the AhR since the ortho chlorines form a rotational barrier that prevents the molecule from assuming a planar conformation. Additionally, *meta* and *para* substitutions also affect the neurotoxic potential of PCBs.^86,98–100^ Several studies have linked these noncoplanar congeners to neurotoxic outcomes despite the inability to interact with the AhR.^44,96,100^ There are several postulated mechanisms through which these noncoplanar PCBs exert their neurotoxic effects, depending on their structure–activity relationships.^11,22,43,79,98,101,102^ Frequently studied cellular mechanisms for PCB neurotoxicity include altered dopamine (DA) signaling,^22,99,100^ disruption of thyroid hormone (TH) signaling,^100^ dysregulation of oxidative stress and reactive oxygen species (ROS),^11,81^ impaired astrocyte functions,^79,103^ and the effects of PCBs on Ca ^(2+)^-homeostasis and inositol phosphates via the Ryanodine receptors (RyR).^77^ PCB52 and its metabolites are among the PCB congeners with high affinity for RyR. The isoforms of RyR are expressed in both excitable and nonexcitable tissues, where they form microsomal Ca^2+^ release channels broadly involved in shaping cellular signaling.

Similarly, the cumulative presence of PCB52 in the ovaries and mammary glands may relate to reproductive system effects and the transfer of PCBs to infants through breast milk. Additionally, the concentration of [^14^C]-PCB52 in the ovaries, mammary glands, and adipose tissues highlights the risk to women of reproductive age, suggesting that PCB52 can accumulate in the body and may affect reproductive health and pregnancy. The extensive maternal tissue distribution data from this study corroborate the biological plausibility of the health effects associated with PCB exposure during pregnancy and underscore the importance of minimizing such exposure to protect maternal and fetal health. They also highlight the need for further research to assess the risks and develop appropriate regulatory measures. The dominance of parent PCB52 over its metabolite in the placenta suggests a potential for bioaccumulation, which could have long-term health implications. Although PCB52 declined rapidly in maternal serum over the 96 h period, its initial dominance relative to metabolites in the maternal serum and sustained dominance in the placenta suggest that a portion of the parent compound persists long enough to cross the placental barrier. This presence may contribute to short-term accumulation in maternal and fetal tissues even in the context of efficient systemic metabolism. Therefore, the potential for bioaccumulation should be interpreted in light of both the timing and tissue-specific distribution of PCB52 and its metabolites. These insights into the toxicokinetics of LC-PCBs justify ongoing research to understand the long-term health consequences of inhalation exposure and to develop regulatory measures that protect sensitive populations.

In line with previous studies, PCB52 accumulates in adipose tissue, representing a potential ongoing source of chronic exposure for both mothers and offspring via breastfeeding.^34,43,62,102,104–107^ Notably, for the first time, we report individual and distinct concentrations of PCB52 in brown and white adipose tissue, with brown adipose tissue attaining the maximum concentration more rapidly than white adipose tissue. Specifically, the concentration of PCB52 in brown adipose was five times higher than in white adipose, and the concentration ratio between these tissues reached unity at 24 h postexposure. Although the proportion of brown adipose tissue in the body is far less than that of white adipose tissue, its role is crucial. While white adipose tissue primarily stores energy, the high mitochondrial content and unique expression of uncoupling protein 1 equips brown adipose tissue to specialize in energy expenditure and thermogenesis.^108–110^ These highlighted roles of brown adipose tissue in body physiology illustrate the significance of understanding the consequences of the higher levels of PCB52 in brown adipose tissue. Moreover, PCBs, a class of endocrine-disrupting chemicals prevalent in the environment, have been linked to chronic diseases such as diabetes and immunotoxicity.^48,111–114^ *In vitro* experiments further demonstrate that PCB mixtures produce toxic effects in mesenchymal stem cells, leading to impaired adipogenesis.^115^ Therefore, the effects of PCBs on adipocytes should be taken into consideration when evaluating potential adverse outcomes resulting from PCB exposure. Furthermore, several studies highlight the adverse effects of endocrine-disrupting chemicals on the biology of mesenchymal stem cells.^111,116^ Obesity is associated with increased mortality and comorbidities, including various metabolic diseases. Therefore, understanding the implications of elevated PCB52 levels in brown adipose tissue is essential, given its physiological significance and potential health effects.

The rapid distribution of PCB52 to the placenta and fetus suggests that PCBs can reach and potentially affect the developing fetus. The findings that PCB52 and its metabolites cross the placental barrier and that the fetus is exposed to similar concentrations as the mother indicate a substantial risk to the developing fetus, which is confirmed by documented adverse health effects of PCB exposure during pregnancy, such as adverse birth outcomes, developmental effects, neurodevelopmental disorders, endocrine disruption, and effects on the reproductive and immune system.^10–14^ Our findings from the ratios of toxicokinetic parameters as a measure of distribution and placental transfer further suggest that the placenta provides a moderate protective shield to the fetus from PCB52 exposure. The contrasting direction between amniotic fluid-to-fetus ratio (<1) and fetus-to-amniotic fluid ratio (>5) suggests a lack of resistance to the moving of PCB52 from amniotic fluid to the fetus and a high resistance in moving from fetus to amniotic fluid, which supports the hypothesis that amniotic fluid serves as an excretory medium during the later stages of development. The concentrations of PCB52 and its metabolites were lower in amniotic fluid than in the maternal plasma and fetus, suggesting comparable fetal exposure.

The rapid metabolism and relatively short biological half-life of less than 2 days in both dams and developing fetuses in this study demonstrate more rapid clearance than that of HC-PCBs. This relatively rapid clearance is reflected in the total elimination of 58.9% at 96 h postexposure, primarily through feces (42.5%) and, to a lesser extent, urine (14.5%). The low-molecular-weight nature of PCB52, along with its possession of two adjacent unsubstituted carbon atoms and two unsubstituted *para*-positions, favors its faster metabolism to phase II metabolites compared with those PCBs lacking these two characteristics.^77,117^ The terminal elimination half-life of PCB52 from this study is comparable to that in previous rodent studies following single oral exposure to a noncommercial PCB mixture containing PCB28, PCB52, PCB77, PCB87, and PCB101,^118^ and following multiple daily exposure to commercial PCB mixture of Kanechlor-300, Kanechlor-400, Kanechlor-500, and Kanechlor-600.^75^ In contrast, another previous study reported a longer half-life for PCB52 (∼10 days) following intravenous administration in male rats.^119^ However, examination of the concentration–time data from the Pu et al. study suggests a half-life of approximately 1.5 to 2 days when their data are fitted to a two-compartmental PK model.^119^ Notably, the study only measured up to 72 h postdosing, which raises questions about the accuracy of the reported half-life.^119^ Measuring to 72 h is insufficient to support a 241 h half-life estimate, as it fails to capture the full terminal elimination phase required for accurate pharmacokinetic estimation.^72,120,121^ This limitation increases the risk of overestimation and misinterpretation of the compound’s persistence. Alternatively, it is possible that this longer half-life value reflects a typographical error or mislabeling between PCB52 and PCB118, as the data for PCB118 do not support a half-life shorter than that of PCB52. These discrepancies highlight the need for cautious interpretation of toxicokinetic parameters and underscore the importance of using well-characterized models for PCB congener comparisons. The existing literature demonstrates that high doses of persistent organic pollutants can alter their kinetic behaviors.^122,123^ Moreover, PCBs exhibit diverse toxicokinetic parameters, including half-lives. HC-PCBs persist for over 90 days, while LC-PCBs have shorter initial half-lives (around 1 to 2 days) in nonpregnant animals.^107,124,125^ Consequently, by providing toxicokinetic data for individual LC-PCB congeners within the low-dose range following lung exposure, our study contributes valuable insights for risk assessment. The difference in the PCB52 biological half-lives may be attributed to several factors that vary from one study to another, including differences in the exposure routes, dosage forms, experimental models, administered dose levels, fasted versus fed animals prior to dosing, and exposure to PCB mixtures versus individual congeners. The metabolism of certain congeners in the PCB mixture is inhibited by other congeners in the mixture, and any nonmetabolized congener present in the mixture can inhibit the metabolism of another individual PCB congener.^126,127^

PCB52 exhibits different disposition and toxicokinetic profiles compared to other LC-PCB congeners.^4,43,61,62,102,104,128–130^ PCB52 is more rapidly metabolized and eliminated in exposed dams than PCB28 and PCB11 in male rats following intratracheal exposure.^61,62^ PCB28 was less extensively metabolized and tended to be retained in the male rat body longer than PCB52 during pregnancy^118,131^ (Adamu et al.,^200^ under review). These differences are consistent with previous reports that PCB congeners with unsubstituted chlorine atoms in vicinal *meta* and *para*-positions, as in PCB52, are readily metabolized by cytochrome P450 enzymes.^66,104,130^

While the rapid elimination of PCB52 in this study, compared to the higher-chlorinated PCBs in previous studies, may reduce the potential for accumulation of the parent compound during pregnancy, the presence of metabolites in the body still poses significant risks due to the presence of metabolites that could affect fetal development. Moreover, the considerable conversion of PCB52 to metabolites within 96 h after exposure, found in maternal serum, fetus, and placenta, highlights the importance of considering these metabolites when assessing the toxicity of PCBs. The presence of metabolites in the fetus and placenta is concerning due to their higher neurotoxicity than the parent PCB52.^79,107,132^ Yamamoto et al. have shown that the major metabolite of tetrachlorobiphenyl (PCB66) is 5 times more toxic than the parent compound.^133^ Furthermore, PCB52 metabolites are more likely to enter the cells due to their structural advantages, resulting in the reported neurotoxicity.^79^ The chlorination pattern and the presence of ortho-substituted chlorines and adjacent unsubstituted *para-* and *meta*-chlorines in PCB52 have been attributed to the higher observed toxicity of its metabolite.^51,79,89,104,127^

Phase II metabolites are the major metabolites of PCB52 following lung exposure. Existing literature provides substantial evidence of the potential toxicity of phase II metabolites of PCBs due to the formation of methyl sulfonyl metabolites that deplete glutathione.^77,79,90,134^ Similar to our findings, recent studies in rats exposed to PCB52 demonstrated formation of a complex mixture of PCB52 metabolites, including sulfated, methoxylated, and dechlorinated forms detected in the lung, liver, and serum, with Phase II metabolites being predominant in these tissues shortly after exposure.^66,130^ In humans, while direct evidence from lung tissue following inhalation exposure is limited, in vitro and in vivo studies using human-relevant models (e.g., HEK293 cells expressing CYP2A6) have demonstrated robust formation of hydroxylated PCB52 metabolites, which are precursors to Phase II conjugates.^3,90,135–137^ Furthermore, sulfate and glucuronide conjugates of PCBs, including PCB52, have been detected in human serum, indicating that Phase II metabolism does occur in humans.^80,90,91^ However, species-specific differences in enzyme expression and involvement,^137^ induction pathways,^90,104^ preferential binding and retention,^51,89^ and microbiome activity^138,139^ suggest that the relative abundance and profile of Phase II metabolites may differ between rodents and humans. For example, rodents exhibit strong induction of CYP2B1 and other enzymes via AhR and PXR activation, while in humans, highly chlorinated PCBs can antagonize PXR, while certain LC-PCBs (like PCB4 and PCB18) can function as partial agonists of the human PXR,^140,141^ suggesting complex interplays potentially influencing phase II detoxification processes. Additionally, the human gut microbiome shows greater variability and capacity to metabolize PCB sulfates compared to rodents.^139^ Therefore, although Phase II metabolites are clearly dominant in rats following lung exposure to PCB52, the same conclusion cannot be definitively extended to humans without further targeted studies. It has been shown that PCB metabolites induce oxidative stress, either directly through the production of reactive oxygen species or indirectly by scavenging antioxidants and inhibiting antioxidant enzymes, thus disturbing cellular redox balance.^77,81,142^ These findings underscore the importance of assessing PCB metabolites when considering the potential behavior and toxicities of PCBs in biological systems, especially during pregnancy.

The detection of PCB52 and metabolites in amniotic fluid within an hour postexposure suggests that amniotic fluid analysis could provide information on both exposure and the potential ongoing risk to the fetus, given the rapid distribution of PCB52 to fetal tissues. The ability to analyze amniotic fluid for PCBs presents a potential tool for prenatal exposure assessment ancillary to amniocentesis procedures. This approach could complement existing maternal biomonitoring strategies and provide early insight into the fetal PCB burden. The idea of utilizing amniotic fluid for biomonitoring of environmental pollutants has been explored for several environmental pollutants.^143–147^ This could be particularly beneficial in the second trimester, during which advanced chromosomal abnormalities or fetal deformities are diagnosed. Such data are invaluable for understanding the potential impact of PCB exposure on both the mother and developing fetus and for informing appropriate public health interventions to minimize the health risks associated with PCB exposure during pregnancy.

The rapid PCB52 metabolism and elimination observed in this study seems at odds with the relatively high levels of PCB52 found in biological matrices and environmental samples as one of the most frequently measured indicator PCBs according to the World Health Organization.^113,148,149^ However, the frequent detection of PCB52 in animal products and human and environmental samples despite its rapid metabolism and elimination suggests that environmental abundance and exposure dynamics play a significant role in its distribution and bioaccumulation. PCB52 is one of the most detected congeners in indoor air, particularly in schools and older buildings, and its physicochemical properties favor volatilization and uptake via inhalation.^5,6,8,42,150^ Repeated or chronic exposure may lead to sustained tissue levels, despite its metabolic lability. Moreover, PCB52 and its metabolites may form covalent adducts or unextractable complexes with macromolecules, contributing to apparent retention. These factors explain the relatively high levels of PCB52 observed in biological samples with its observed bioaccumulation and support its relevance in exposure assessments. These factors also point to the need for toxicokinetic models that account for both parent compound and metabolite behavior across exposure routes.

Our findings shed light on the potential health risks associated with exposure to LC-PCBs and help inform risk assessment. The new ADME profiles of PCB52 from this study provide a basis for developing toxicokinetic models and toxicity evaluations relevant to human exposure. The relationship between the new toxicokinetic profiles and the adverse effects of PCBs during pregnancy warrants further exploration. Overall, these new insights can guide the development of inhalation-based regulatory standards and protective measures to minimize the health risks associated with PCB exposure.

## 5 Conclusion and Practical Implications

Our study simulating inhalation exposure demonstrates PCB52 accumulation in the maternal brain, ovaries, and mammary glands, suggesting a higher risk to women of reproductive age. We provide new insights into the disposition of PCB52 in maternal tissues and the developing fetus following lung exposure. Our findings confirm that PCB52 is efficiently absorbed and distributed throughout the body, reaching the placenta, developing fetuses, and amniotic fluid. Our research demonstrates for the first time that lung exposure to PCB52 leads to the formation of several major PCB52 metabolites in the placenta and developing fetuses, including hydroxylated, methoxylated, and conjugated PCB52 metabolites, which are known to be toxic.

The absence of established inhalation-based reference concentrations (RfC) for PCBs by regulatory agencies highlights a critical gap in our understanding of the risks associated with airborne PCB exposure. Our study helps close this gap by characterizing the tissue distribution, maternal–fetal transfer, and toxicokinetic parameters following acute PCB52 lung exposure. The results support the urgent need for developing toxicokinetic models and conducting toxicity assessments of airborne PCBs, particularly PCB52 and its metabolites, due to their prevalence in indoor and outdoor environments such as schools and demonstrated toxicological effects following exposure to LC-PCBs and their metabolites.^23,79,87,92,151,152^ By providing empirical data on the presence of PCB52 and its metabolites in target tissues, our study supports efforts to regulate PCB levels in school buildings, ensuring that indoor air quality safeguards the health of sensitive populations, especially during subacute to chronic inhalation exposure. Regulatory agencies can utilize these findings to inform risk assessments and establish safety standards for human exposure to airborne PCBs, ultimately contributing to the enhancement of public health.

## Supplementary Material



## Notes

Percent tissue distribution of [^14^C]-PCB52 over time following lung exposure (Table S1); toxicokinetic parameters derived from total [^14^C]-PCB52 concentration–time data (Table S2); schematic illustration of air and excreta sampling during the postexposure observatory period (Figure S1); and Pearson correlation coefficients of [^14^C]-PCB52 concentration in maternal tissues, digestive matter, developing fetus, and amniotic fluids following intratracheal dosing at early pregnancy (PDF)

## References

[1] Melymuk L., Blumenthal J., Sáňka Oe., Shu-Yin A., Singla V., Šebková Ki., Pullen Fedinick K., Diamond M. L. (2022). Persistent problem: global challenges to managing PCBs. Environ. Sci. Technol..

[2] Harrad S., Ibarra C., Diamond M., Melymuk L., Robson M., Douwes J., Roosens L., Dirtu A. C., Covaci A. (2008). Polybrominated
diphenyl ethers in domestic indoor dust from Canada, New Zealand,
United Kingdom and United States. Environ. Int..

[3] Thorne P., Ampleman M., Hu X., Adamcakova-Dodd A., Hornbuckle K. (2015). Uptake of inhaled polychlorinated biphenyls (PCBs)
in a human longitudinal cohort study and animal inhalation studies. Eur. Respiratory Soc..

[4] Hu X., Adamcakova-Dodd A., Lehmler H. J., Hu D., Kania-Korwel I., Hornbuckle K. C., Thorne P. S. (2010). Time course of congener uptake and
elimination in rats after short-term inhalation exposure to an airborne
polychlorinated biphenyl (PCB) mixture. Environ.
Sci. Technol..

[5] Hammel S. C., Andersen H. V., Knudsen L. E., Frederiksen M. (2023). Inhalation
and dermal absorption as dominant pathways of PCB exposure for residents
of contaminated apartment buildings. Int. J.
Hyg Environ. Health.

[6] Marek R. F., Thorne P. S., Herkert N. J., Awad A. M., Hornbuckle K. C. (2017). Airborne
PCBs and OH-PCBs inside and outside urban and rural US schools. Environ. Sci. Technol..

[7] Marek R. F., Thorne P. S., DeWall J., Hornbuckle K. C. (2014). Variability
in PCB and OH-PCB serum levels in children and their mothers in urban
and rural US communities. Environ. Sci. Technol..

[8] Ampleman M.
D., Martinez A., DeWall J., Rawn D. F., Hornbuckle K. C., Thorne P. S. (2015). Inhalation and dietary exposure to PCBs in urban and
rural cohorts via congener-specific measurements. Environ. Sci. Technol..

[9] Liebl B., Schettgen T., Kerscher G., Broding H.-C., Otto A., Angerer J., Drexler H. (2004). Evidence for increased internal exposure
to lower chlorinated polychlorinated biphenyls (PCB) in pupils attending
a contaminated school. Int. J. Hyg. Environ.
Health.

[10] Grandjean P., Landrigan P. J. (2006). Developmental neurotoxicity of industrial chemicals. Lancet.

[11] Berghuis S. A., Bos A. F., Sauer P. J., Roze E. (2015). Developmental neurotoxicity
of persistent organic pollutants: an update on childhood outcome. Arch. Toxicol..

[12] Bergonzi R., De Palma G., Specchia C., Dinolfo M., Tomasi C., Frusca T., Apostoli P. (2011). Persistent organochlorine
compounds
in fetal and maternal tissues: evaluation of their potential influence
on several indicators of fetal growth and health. Sci. Total Environ..

[13] Kofoed A. B., Deen L., Hougaard K. S., Petersen K. U., Meyer H. W., Pedersen E. B., Ebbehøj N. E., Heitmann B. L., Bonde J. P., Tøttenborg S. S. (2021). Maternal
exposure to airborne polychlorinated biphenyls
(PCBs) and risk of adverse birth outcomes. Eur.
J. Epidemiol..

[14] Roze E., Meijer L., Bakker A., Van Braeckel K. N., Sauer P. J., Bos A. F. (2009). Prenatal exposure to organohalogens,
including brominated flame retardants, influences motor, cognitive,
and behavioral performance at school age. Environ.
Health Perspect..

[15] Goodman D. R., James R. C., Harbison R. D. (1982). Placental
toxicology. Food Chem. Toxicol..

[16] Furukawa S., Tsuji N., Sugiyama A. (2019). Morphology
and physiology of rat
placenta for toxicological evaluation. J. Toxicol.
Pathol..

[17] Furukawa S., Hayashi S., Usuda K., Abe M., Hagio S., Ogawa I. (2011). Toxicological pathology in the rat placenta. J. Toxicol. Pathol..

[18] Yang X., Liu Y., Liu S., Zheng P., Bai X., Ma L. Q., Liu W. (2023). Prenatal exposure to 209 PCBs in mother-infant pairs from two cities
in China: Levels, congener profiles, and transplacental transfer. Chemosphere.

[19] Zhang X., Cheng X., Lei B., Zhang G., Bi Y., Yu Y. (2021). A review of the transplacental
transfer of persistent halogenated
organic pollutants: Transfer characteristics, influential factors,
and mechanisms. Environ. Int..

[20] Fukata H., Omori M., Osada H., Todaka E., Mori C. (2005). Necessity
to measure PCBs and organochlorine pesticide concentrations in human
umbilical cords for fetal exposure assessment. Environ. Health Perspect..

[21] Guvenius D. M., Aronsson A., Ekman-Ordeberg G., Bergman A., Norén K. (2003). Human prenatal
and postnatal exposure to polybrominated diphenyl ethers, polychlorinated
biphenyls, polychlorobiphenylols, and pentachlorophenol. Environ. Health Perspect..

[22] Pessah I. N., Hansen L. G., Albertson T. E., Garner C. E., Ta T. A., Do Z., Kim K. H., Wong P. W. (2006). Structure– activity relationship
for noncoplanar polychlorinated biphenyl congeners toward the ryanodine
receptor-Ca2+ channel complex type 1 (RyR1). Chem. Res. Toxicol..

[23] Zhao D., Wang Q., Zhou W.-T., Wang L.-B., Yu H., Zhang K.-K., Chen L.-J., Xie X.-L. (2020). PCB52 exposure alters
the neurotransmission ligand-receptors in male offspring and contributes
to sex-specific neurodevelopmental toxicity. Environ. Pollut..

[24] Park H. Y., Hertz-Picciotto I., Sovcikova E., Kocan A., Drobna B., Trnovec T. (2010). Neurodevelopmental
toxicity of prenatal polychlorinated
biphenyls (PCBs) by chemical structure and activity: a birth cohort
study. Environ. Health.

[25] Brun N. R., Panlilio J. M., Zhang K., Zhao Y., Ivashkin E., Stegeman J. J., Goldstone J. V. (2021). Developmental
exposure to non-dioxin-like
polychlorinated biphenyls promotes sensory deficits and disrupts dopaminergic
and GABAergic signaling in zebrafish. Commun.
Biol..

[26] Loch-Caruso R. (2002). Uterine muscle
as a potential target of polychlorinated biphenyls during pregnancy. Int. J. Hyg. Environ. Health.

[27] Brant K. A., Caruso R. L. (2006). PCB 50 stimulates
release of arachidonic acid and prostaglandins
from late gestation rat amnion fibroblast cells. Reprod. Toxicol..

[28] Moenning J.-L., Numata J., Bloch D., Jahnke A., Schafft H. A., Spolders M., Lüth A., Lahrssen-Wiederholt M., Schulz K. (2023). Transfer and toxicokinetic modeling
of non-dioxin-like
polychlorinated biphenyls (ndl-PCBs) into accidentally exposed dairy
cattle and their calves - A case report. Environ.
Toxicol. Pharmacol..

[29] Lehmann G. M., LaKind J. S., Davis M. H., Hines E. P., Marchitti S. A., Alcala C., Lorber M. (2018). Environmental Chemicals
in Breast
Milk and Formula: Exposure and Risk Assessment Implications. Environ. Health Perspect.

[30] Kawashiro Y., Fukata H., Omori-Inoue M., Kubonoya K., Jotaki T., TAKIGAM H., Sakai S.-I., Mori C. (2008). Perinatal exposure
to brominated flame retardants and polychlorinated biphenyls in Japan. Endocr. J..

[31] Covaci A., Jorens P., Jacquemyn Y., Schepens P. (2002). Distribution of PCBs
and organochlorine pesticides in umbilical cord and maternal serum. Sci. Total Environ..

[32] Vizcaino E., Grimalt J. O., Fernández-Somoano A., Tardon A. (2014). Transport
of persistent organic pollutants across the human placenta. Environ. Int..

[33] Soechitram S. D., Athanasiadou M., Hovander L., Bergman Å., Sauer P. J. J. (2004). Fetal
exposure to PCBs and their hydroxylated metabolites in a Dutch cohort. Environ. Health Perspect..

[34] Gómara B., Herrero L., Ramos J. J., Mateo J., Fernandez M., García J. F., González M. J. (2007). Distribution of polybrominated diphenyl
ethers in human umbilical cord serum, paternal serum, maternal serum,
placentas, and breast milk from Madrid population, Spain. Environ. Sci. Technol..

[35] Walkowiak J., Wiener J.-A., Fastabend A., Heinzow B., Krämer U., Schmidt E., Steingrüber H.-J., Wundram S., Winneke G. (2001). Environmental
exposure to polychlorinated biphenyls and quality of the home environment:
effects on psychodevelopment in early childhood. Lancet.

[36] Santos A. S. E., Moreira J. C., Rosa A. C. S., Câmara V. M., Azeredo A., Asmus C. I. R. F., Meyer A. (2023). Persistent organic
pollutant levels in maternal and cord blood plasma and breast milk:
results from the Rio Birth Cohort Pilot Study of Environmental Exposure
and Childhood Development (PIPA Study). Int.
J. Environ. Res. Public Health.

[37] Kania-Korwel I., Barnhart C. D., Lein P. J., Lehmler H. J. (2015). Effect of pregnancy
on the disposition of 2,2′,3,5′,6-pentachlorobiphenyl
(PCB 95) atropisomers and their hydroxylated metabolites in female
mice. Chem. Res. Toxicol..

[38] Wu C., Du X., Liu H., Chen X., Ge K., Meng R., Zhang Z., Zhang H. (2024). Advances in polychlorinated biphenyls-induced
female reproductive toxicity. Sci. Total Environ..

[39] Schulz B., Carlson L. M., Christensen K., Weitekamp C. A., Marek R. F., Martinez A., Hornbuckle K. C., Lehmann G. M. (2024). Comprehensive compilation of congener profiles to support
health assessment of environmental exposures to polychlorinated biphenyl
mixtures. Environ. Res..

[40] Klocke C., Lein P. J. (2020). Evidence Implicating
Non-Dioxin-Like Congeners as the
Key Mediators of Polychlorinated Biphenyl (PCB) Developmental Neurotoxicity. Int. J. Mol. Sci..

[41] Carlson, L. M. ; Christensen, K. ; Lehmann, G. M. ; Arzuaga, X. ; Coffman, E. ; Shaffer, R. M. ; Yost, E. E. EPA IRIS Assessments. In A Systematic Evidence Map of Noncancer Health Endpoints and Exposures to Polychlorinated Biphenyl (PCB) Mixtures; U.S. Environmental Protection Agency, 2023. 39607947

[42] Weitekamp C. A., Phillips L. J., Carlson L. M., DeLuca N. M., Cohen
Hubal E. A., Lehmann G. M. (2021). A state-of-the-science review of
polychlorinated biphenyl exposures at background levels: relative
contributions of exposure routes. Sci. Total
Environ..

[43] Wang H., Adamcakova-Dodd A., Flor S., Gosse L., Klenov V. E., Stolwijk J. M., Lehmler H. J., Hornbuckle K. C., Ludewig G., Robertson L. W., Thorne P. S. (2020). Comprehensive Subchronic
Inhalation Toxicity Assessment of an Indoor School Air Mixture of
PCBs. Environ. Sci. Technol..

[44] Shain W., Bush B., Seegal R. (1991). Neurotoxicity
of polychlorinated
biphenyls: structure-activity relationship of individual congeners. Toxicol. Appl. Pharmacol..

[45] Paranjape N., Dean L. E., Martinez A., Tjalkens R. B., Lehmler H.-J., Doorn J. A. (2023). Structure–Activity
Relationship of Lower Chlorinated
Biphenyls and Their Human-Relevant Metabolites for Astrocyte Toxicity. Chem. Res. Toxicol..

[46] Carrera A. R. M., Eleazar E. G., Caparanga A. R., Tayo L. L. (2024). Theoretical Studies
on the Quantitative Structure–Toxicity Relationship of Polychlorinated
Biphenyl Congeners Reveal High Affinity Binding to Multiple Human
Nuclear Receptors. Toxics.

[47] Plíšková M., Vondrácek J., Canton R. F., Nera J., Kocan A., Petrík J., Trnovec T., Sanderson T., van den Berg M., Machala M. (2005). Impact of polychlorinated biphenyls
contamination on estrogenic activity in human male serum. Environ. Health Perspect.

[48] Lan T., Liu B., Bao W., Thorne P. S. (2023). Identification of PCB congeners and
their thresholds associated with diabetes using decision tree analysis. Sci. Rep..

[49] Lehmler H.-J., Robertson L. W. (2001). Synthesis of hydroxylated PCB metabolites
with the
Suzuki-coupling. Chemosphere.

[50] Li X., Holland E. B., Feng W., Zheng J., Dong Y., Pessah I. N., Duffel M. W., Robertson L. W., Lehmler H.-J. (2018). Authentication of synthetic environmental
contaminants
and their (bio) transformation products in toxicology: polychlorinated
biphenyls as an example. Environ. Sci. Pollut.
Res..

[51] Rodriguez E. A., Li X., Lehmler H.-J., Robertson L. W., Duffel M. W. (2016). Sulfation of Lower
Chlorinated Polychlorinated Biphenyls Increases Their Affinity for
the Major Drug-Binding Sites of Human Serum Albumin. Environ. Sci. Technol..

[52] Lehmler H.-J., Robertson L. W. (2001). Synthesis
of polychlorinated biphenyls (PCBs) using
the Suzuki-coupling. Chemosphere.

[53] Loryan I., Sinha V., Mackie C., Van Peer A., Drinkenburg W., Vermeulen A., Morrison D., Monshouwer M., Heald D., Hammarlund-Udenaes M. (2014). Mechanistic
understanding of brain
drug disposition to optimize the selection of potential neurotherapeutics
in drug discovery. Pharm. Res..

[54] Nonaka S., Hough C. J., Chuang D.-M. (1998). Chronic
lithium treatment robustly
protects neurons in the central nervous system against excitotoxicity
by inhibiting N-methyl-D-aspartate receptor-mediated calcium influx. Proc. Natl. Acad. Sci. U. S. A..

[55] Reddy R., Pillay V., Baijnath S., Singh S. D., Ramdin S., Naicker T., Govender N. (2022). Mating success
of timed pregnancies
in Sprague Dawley rats: Considerations for execution. Reprod. Biol..

[56] Semple B. D., Blomgren K., Gimlin K., Ferriero D. M., Noble-Haeusslein L. J. (2013). Brain development
in rodents and humans: Identifying benchmarks of maturation and vulnerability
to injury across species. Prog. Neurobiol..

[57] Yager J. Y., Ashwal S. (2009). Animal Models of Perinatal
Hypoxic-Ischemic Brain Damage. Pediatr. Neurol..

[58] Clancy B., Finlay B. L., Darlington R. B., Anand K. J. S. (2007). Extrapolating
brain development from experimental species to humans. Neurotoxicology.

[59] Faroon, O. M. ; Samuel Keith, L. ; Smith-Simon, C. ; De Rosa, C. T. ; Organization, W. H. Polychlorinated Biphenyls: Human Health Aspects; World Health Organization, 2003.

[60] Doull J. (2003). The “Red
Book” and other risk assessment milestones. Hum. Ecol. Risk Assess.: Int. J..

[61] Hu X., Adamcakova-Dodd A., Lehmler H.-J., Hu D., Hornbuckle K., Thorne P. S. (2012). Subchronic inhalation exposure study of an airborne
polychlorinated biphenyl mixture resembling the Chicago ambient air
congener profile. Environ. Sci. Technol..

[62] Hu X., Adamcakova-Dodd A., Thorne P. S. (2014). The fate of inhaled (14)­C-labeled
PCB11 and its metabolites in vivo. Environ.
Int..

[63] Patton J. S., Fishburn C. S., Weers J. G. (2004). The lungs as a portal of entry for
systemic drug delivery. Proc. Am. Thorac Soc..

[64] Borghardt J. M., Weber B., Staab A., Kloft C. (2015). Pharmacometric Models
for Characterizing the Pharmacokinetics of Orally Inhaled Drugs. AAPS J..

[65] Schanker L. S., Mitchell E. W., Brown R. A. (1986). Species comparison
of drug absorption from the lung after aerosol inhalation or intratracheal
injection. Drug Metab. Dispos..

[66] Bullert A. J., Li X., Gautam B., Wang H., Adamcakova-Dodd A., Wang K., Thorne P. S., Lehmler H.-J. (2024). Distribution of
2,2′,5,5′-Tetrachlorobiphenyl (PCB52) Metabolites in
Adolescent Rats after Acute Nose-Only Inhalation Exposure. Environ. Sci. Technol..

[67] Penner N., Xu L., Prakash C. (2012). Radiolabeled
absorption, distribution, metabolism,
and excretion studies in drug development: why, when, and how?. Chem. Res. Toxicol..

[68] Temerin L. A. (1985). Size, function,
and life history. By W.A. Calder III. Cambridge: Harvard University
Press. 1984. xii + 431 pp., figures, tables, appendices, index. $32.50
(cloth). Am. J. Phys. Anthropol..

[69] Shen H., Han J., Guan R., Cai D., Zheng Y., Meng Z., Chen Q., Li J., Wu Y. (2022). Use of different endpoints
to determine the bioavailability of polychlorinated dibenzo-p-dioxins/furans
(PCDD/Fs) and polychlorinated biphenyls (PCBs) in Sprague–Dawley
rats. Sci. Rep..

[70] Holder D. J. (2001). Comments
on Nedelman and Jia’s extension of Satterthwaite’s approximation
applied to pharmacokinetics. J. Biopharm. Stat..

[71] Nedelman J. R., Jia X. (1998). An extension of satterth waite’s approximation applied to
pharmacokinetics. J. Biopharm. Stat..

[72] Bi Y., Deng J., Murry D. J., An G. (2016). A whole-body physiologically
based pharmacokinetic model of gefitinib in mice and scale-up to humans. AAPS J..

[73] Gallo J. M., Lam F. C., Perrier D. G. (1987). Area method for
the estimation of
partition coefficients for physiological pharmacokinetic models. J. Pharmacokinet. Biopharm..

[74] Albro P. W., Fishbein L. (1972). Intestinal absorption
of polychlorinated biphenyls
in rats. Bull. Environ. Contam. Toxicol..

[75] Tanabe S., Nakagawa Y., Tatsukawa R. (1981). Absorption
Efficiency and Biological
Half-Life of Individual Chlorobiphenyls in Rats Treated with Kanechlor
Products. Agric. Biol. Chem..

[76] Haraguchi K., Koga N., Kato Y. (2005). Comparative
metabolism of polychlorinated
biphenyls and tissue distribution of persistent metabolites in rats,
hamsters, and guinea pigs. Drug Metab. Dispos..

[77] Liu J., Tan Y., Song E., Song Y. (2020). A Critical Review of Polychlorinated
Biphenyls Metabolism, Metabolites, and Their Correlation with Oxidative
Stress. Chem. Res. Toxicol..

[78] ATSDR . Toxicological Profile for Polychlorinated Biphenyls (PCBs); Agency for Toxic Substances and Disease Registry: Atlanta, GA, 2000; Vol. 30333. 36888731

[79] Paranjape N., Dean L. E., Martinez A., Tjalkens R. B., Lehmler H. J., Doorn J. A. (2023). Structure-Activity
Relationship of Lower Chlorinated
Biphenyls and Their Human-Relevant Metabolites for Astrocyte Toxicity. Chem. Res. Toxicol..

[80] Li X., Hefti M. M., Marek R. F., Hornbuckle K. C., Wang K., Lehmler H. J. (2022). Assessment of Polychlorinated
Biphenyls
and Their Hydroxylated Metabolites in Postmortem Human Brain Samples:
Age and Brain Region Differences. Environ. Sci.
Technol..

[81] Bullert A. J., Wang H., Valenzuela A. E., Neier K., Wilson R. J., Badley J. R., LaSalle J. M., Hu X., Lein P. J., Lehmler H.-J. (2024). Interactions of Polychlorinated Biphenyls and Their
Metabolites with the Brain and Liver Transcriptome of Female Mice. ACS Chem. Neurosci..

[82] Seelbach M., Chen L., Powell A., Choi Y. J., Zhang B., Hennig B., Toborek M. (2010). Polychlorinated
biphenyls disrupt
blood-brain barrier integrity and promote brain metastasis formation. Environ. Health Perspect.

[83] Selvakumar K., Prabha R. L., Saranya K., Bavithra S., Krishnamoorthy G., Arunakaran J. (2013). Polychlorinated
biphenyls impair blood–brain
barrier integrity via disruption of tight junction proteins in cerebrum,
cerebellum and hippocampus of female Wistar rats:Neuropotential role
of quercetin. Hum. Exp. Toxicol..

[84] Shimada T., Imai Y., Sato R. (1981). Covalent binding
of polychlorinated
biphenyls to proteins by reconstituted monooxygenase system containing
cytochrome P-450. Chem.-Biol. Interact..

[85] Shimada T., Sato R. (1980). Covalent binding of polychlorinated biphenyls to rat liver microsomes
in vitro: Nature of reactive metabolites and target macromolecules. Toxicol. Appl. Pharmacol..

[86] Holland E. B., Feng W., Zheng J., Dong Y., Li X., Lehmler H.-J., Pessah I. N. (2017). An extended
structure–activity
relationship of nondioxin-like PCBs evaluates and supports modeling
predictions and identifies picomolar potency of PCB 202 towards ryanodine
receptors. Toxicol. Sci..

[87] Pessah I. N., Lein P. J., Seegal R. F., Sagiv S. K. (2019). Neurotoxicity of
polychlorinated biphenyls and related organohalogens. Acta Neuropathol..

[88] Do Y., Lee D. K. (2012). Effects of polychlorinated
biphenyls on the development
of neuronal cells in growth period; structure-activity relationship. Exp. Neurobiol..

[89] Rodriguez E. A., Vanle B. C., Doorn J. A., Lehmler H. J., Robertson L. W., Duffel M. W. (2018). Hydroxylated and
sulfated metabolites of commonly observed
airborne polychlorinated biphenyls display selective uptake and toxicity
in N27, SH-SY5Y, and HepG2 cells. Environ. Toxicol.
Pharmacol..

[90] Duffel M. W., Lehmler H. J. (2024). Complex roles for sulfation in the toxicities of polychlorinated
biphenyls. Crit. Rev. Toxicol..

[91] Zhang D., Saktrakulkla P., Marek R. F., Lehmler H.-J., Wang K., Thorne P. S., Hornbuckle K. C., Duffel M. W. (2022). PCB Sulfates in
Serum from Mothers and Children in Urban and Rural U.S. Communities. Environ. Sci. Technol..

[92] Bullert A. J., Wang H., Linahon M. J., Chimenti M. S., Adamcakova-Dodd A., Li X., Dailey M. E., Klingelhutz A. J., Ankrum J. A., Stevens H. E. (2024). Effects
of 28-day nose-only inhalation of PCB52 (2,2′,5,5′-Tetrachlorobiphenyl)
on the brain transcriptome. Toxicology.

[93] IARC . IARC Monographs on the Evaluation of Carcinogenic Risks to Humans Polychlorinated Dibenzo-Para-Dioxins and Polychlorinated Dibenzofurans 1997. PMC5366851 9379504

[94] Pradeep P., Carlson L. M., Judson R., Lehmann G. M., Patlewicz G. (2019). Integrating
data gap filling techniques: A case study predicting TEFs for neurotoxicity
TEQs to facilitate the hazard assessment of polychlorinated biphenyls. Regul. Toxicol. Pharmacol..

[95] Ruiz P., Faroon O., Moudgal C., Hansen H., De Rosa C., Mumtaz M. (2008). Prediction of the health
effects of polychlorinated
biphenyls (PCBs) and their metabolites using quantitative structure–activity
relationship (QSAR). Toxicol. Lett..

[96] Seegal R. F., Bush B., Shain W. (1991). Neurotoxicology
of ortho-substituted
polychlorinated biphenyls. Chemosphere.

[97] Stenberg M., Hamers T., Machala M., Fonnum F., Stenius U., Lauy A. A., van Duursen M. B. M., Westerink R. H. S., Fernandes E. C. A., Andersson P. L. (2011). Multivariate
toxicity profiles and QSAR modeling of non-dioxin-like PCBs - An investigation
of in vitro screening data from ultra-pure congeners. Chemosphere.

[98] Kodavanti P., Tilson H. (1997). Structure-activity
relationships of potentially neurotoxic
PCB congeners in the rat. Neurotoxicology.

[99] Seegal R.
F., Brosch K. O., Bush B. (1986). Polychlorinated biphenyls produce
regional alterations of dopamine metabolism in rat brain. Toxicol. Lett..

[100] Seegal R. F., Bush B., Shain W. (1990). Lightly chlorinated
ortho-substituted PCB congeners decrease dopamine in nonhuman primate
brain and in tissue culture. Toxicol. Appl.
Pharmacol..

[101] Rayne S., Forest K. (2010). Quantitative structure-activity relationship
(QSAR) studies for predicting activation of the ryanodine receptor
type 1 channel complex (RyR1) by polychlorinated biphenyl (PCB) congeners. J. Environ. Sci. Health, Part A.

[102] Wang H., Adamcakova-Dodd A., Lehmler H.-J., Hornbuckle K. C., Thorne P. S. (2022). Toxicity assessment
of 91-day repeated inhalation exposure
to an indoor school air mixture of PCBs. Environ.
Sci. Technol..

[103] Sethi S., Keil K. P., Chen H., Hayakawa K., Li X., Lin Y., Lehmler H. J., Puschner B., Lein P. J. (2017). Detection
of 3,3′-Dichlorobiphenyl in Human Maternal Plasma and Its Effects
on Axonal and Dendritic Growth in Primary Rat Neurons. Toxicol. Sci..

[104] Grimm F. A., Hu D., Kania-Korwel I., Lehmler H. J., Ludewig G., Hornbuckle K. C., Duffel M. W., Bergman Å., Robertson L. W. (2015). Metabolism
and metabolites of polychlorinated biphenyls. Crit. Rev. Toxicol..

[105] Louis C., Tinant G., Mignolet E., Thomé J. P., Debier C. (2014). PCB-153 shows different dynamics of mobilisation from
differentiated rat adipocytes during lipolysis in comparison with
PCB-28 and PCB-118. PLoS One.

[106] Lauby-Secretan B., Loomis D., Grosse Y., El Ghissassi F., Bouvard V., Benbrahim-Tallaa L., Guha N., Baan R., Mattock H., Straif K. (2013). Carcinogenicity of polychlorinated
biphenyls and polybrominated biphenyls. Lancet
Oncol..

[107] Matthews H., Anderson M. (1975). Effect of chlorination
on the distribution
and excretion of polychlorinated biphenyls. Drug Metab. Dispos..

[108] Townsend K., Tseng Y. H. (2012). Brown adipose tissue: Recent insights
into development, metabolic function and therapeutic potential. Adipocyte.

[109] Nakagami H. (2013). The mechanism
of white and brown adipocyte differentiation. Diabetes Metab J..

[110] Jung S. M., Sanchez-Gurmaches J., Guertin D. A. (2019). Brown Adipose Tissue
Development and Metabolism. Handb. Exp. Pharmacol..

[111] Di Gregorio I., Busiello R. A., Burgos
Aceves M. A., Lepretti M., Paolella G., Lionetti L. (2019). Environmental
Pollutants
Effect on Brown Adipose Tissue. Front. Physiol..

[112] Bateman M. E., Strong A. L., McLachlan J. A., Burow M. E., Bunnell B. A. (2017). The Effects of Endocrine Disruptors
on Adipogenesis and Osteogenesis in Mesenchymal Stem Cells: A Review. Front. Endocrinol..

[113] Li P., Xu Y., Li Z., Cheng X., Jia C., Zhang S., An J., Zhang X., Yan Y., He M. (2023). Association between
polychlorinated biphenyls exposure and incident
type 2 diabetes mellitus: A nested case-control study. Environ. Res..

[114] Wang S. L., Tsai P. C., Yang C. Y., Guo Y. L. (2008). Increased
risk of diabetes and polychlorinated biphenyls and dioxins: a 24-year
follow-up study of the Yucheng cohort. Diabetes
Care.

[115] Behan-Bush R. M., Liszewski J. N., Schrodt M. V., Vats B., Li X., Lehmler H.-J., Klingelhutz A. J., Ankrum J. A. (2023). Toxicity Impacts
on Human Adipose Mesenchymal Stem/Stromal Cells Acutely Exposed to
Aroclor and Non-Aroclor Mixtures of Polychlorinated Biphenyl. Environ. Sci. Technol..

[116] Wang B., Steinberg G. R. (2022). Environmental
toxicants, brown adipose
tissue, and potential links to obesity and metabolic disease. Curr. Opin. Pharmacol..

[117] Schnellmann R. G., Volp R. F., Putnam C. W., Sipes I. G. (1984). The hydroxylation,
dechlorination, and glucuronidation of 4,4′-dichlorobiphenyl
(4-DCB) by human hepatic microsomes. Biochem.
Pharmacol..

[118] Öberg M., Sjödin A., Casabona H., Nordgren I., Klasson-Wehler E., Håkansson H. (2002). Tissue distribution and half-lives
of individual polychlorinated biphenyls and serum levels of 4-hydroxy-2,
3, 3, 4, 5-pentachlorobiphenyl in the rat. Toxicol.
Sci..

[119] Pu X., Lee L. S., Galinsky R. E., Carlson G. P. (2006). Bioavailability
of 2,3′,4,4′,5-pentachlorobiphenyl (PCB118) and 2,2′,5,5′-tetrachlorobiphenyl
(PCB52) from soils using a rat model and a physiologically based extraction
test. Toxicology.

[120] Colucci P., Turgeon J., Ducharme M. P. (2011). How critical
is
the duration of the sampling scheme for the determination of half-life,
characterization of exposure and assessment of bioequivalence. J. Pharm. Pharm. Sci..

[121] Jeong Y.-S., Jusko W. J. (2022). Determinants of
Biological Half-Lives
and Terminal Slopes in Physiologically Based Pharmacokinetic Systems:
Assessment of Limiting Conditions. AAPS J..

[122] Santostefano M. J., Ross D. G., Savas U., Jefcoate C. R., Birnbaum L. S. (1997). Differential
Time–Course and Dose–Response
Relationships of TCDD-Induced CYP1B1, CYP1A1, and CYP1A2 Proteins
in Rats. Biochem. Biophys. Res. Commun..

[123] De Jongh J., Nieboer R., Schröders I., Seinen W., Van den
Berg M. (1993). Toxicokinetic mixture interactions
between chlorinated aromatic hydrocarbons in the liver of the C57BL/6J
mouse: 2. polychlorinated dibenzo-p-dioxins (PCDDs), dibenzofurans
(PCDFs) and biphenyls (PCBs). Arch. Toxicol..

[124] Lutz R. J., Dedrick R., Tuey D., Sipes I., Anderson M., Matthews H. (1984). Comparison of the pharmacokinetics
of several polychlorinated biphenyls in mouse, rat, dog, and monkey
by means of a physiological pharmacokinetic model. Drug Metab. Dispos..

[125] Matthews H. B., Tuey D. B. (1980). The effect of chlorine
position on
the distribution and excretion of four hexachlorobiphenyl isomers. Toxicol. Appl. Pharmacol..

[126] Tsiaoussis J., Antoniou M. N., Koliarakis I., Mesnage R., Vardavas C. I., Izotov B. N., Psaroulaki A., Tsatsakis A. (2019). Effects of single and combined toxic exposures on the
gut microbiome: Current knowledge and future directions. Toxicol. Lett..

[127] Mills R. A., Millis C. D., Dannan G. A., Guengerich F. P., Aust S. D. (1985). Studies on the structure-activity relationships for
the metabolism of polybrominated biphenyls by rat liver microsomes. Toxicol. Appl. Pharmacol..

[128] Batterman S. A., Chernyak S., Su F. C. (2016). Measurement
and
Comparison of Organic Compound Concentrations in Plasma, Whole Blood,
and Dried Blood Spot Samples. Front. Genet..

[129] Zhang C.-Y., Flor S., Ruiz P., Ludewig G., Lehmler H.-J. (2021). Characterization of the Metabolic
Pathways of 4-Chlorobiphenyl
(PCB3) in HepG2 Cells Using the Metabolite Profiles of Its Hydroxylated
Metabolites. Environ. Sci. Technol..

[130] Bullert A., Li X., Chunyun Z., Lee K., Pulliam C. F., Cagle B. S., Doorn J. A., Klingelhutz A. J., Robertson L. W., Lehmler H.-J. (2023). Disposition and metabolomic effects
of 2,2′,5,5′-tetrachlorobiphenyl in female rats following
intraperitoneal exposure. Environ. Toxicol.
Pharmacol..

[131] Moir D., Viau A., Chu I., Wehler E. K., Mörck A., Bergman A. (1996). Tissue distribution, metabolism,
and excretion of 2,4,4′-trichlorobiphenyl (CB-28) in the rat. Toxicol. Ind. Health.

[200] Adamu Y., Brandon N. M., Adamcakova-Dodd A., Wang H., Thorne P. S. (2025). Whole-Body Disposition and Metabolism
of [14C]-2,4,4′-Trichlorobiphenyl (PCB28) Following Lung Administration
in Rats. Environ. Sci.Technol..

[132] Stadnicki S. S., Allen J. R. (1979). Toxicity of 2,2′,5,5′-tetrachlorobiphenyl
and its metabolites, 2,2′,5,5′-tetrachlorobiphenyl-3,4-oxide
and 2,2′,5,5′-tetrachlorobiphenyl-4-o1 to cultured cellsin
vitro. Bull. Environ. Contam. Toxicol..

[133] Yoshimura H., Yamamoto Ha. (1975). A novel route of excretion of 2,
4, 3′, 4′-tetrachlorobiphenyl in rats. Bull. Environ. Contam. Toxicol..

[134] Bakke J., Gustafsson J.-Å. (1984). Mercapturic
acid pathway metabolites
of xenobiotics: generation of potentially toxic metabolites during
enterohepatic circulation. Trends Pharmacol.
Sci..

[135] Allen J. R., Cartens L. A., Abrahamson L. J., Marlar R. J. (1975). Responses of rats
and nonhuman primates to 2,5,2′,5′-tetrachlorobiphenyl. Environ. Res..

[136] Uwimana E., Cagle B., Yeung C., Li X., Patterson E. V., Doorn J. A., Lehmler H.-J. (2019). Atropselective Oxidation
of 2,2′,3,3′,4,6′-Hexachlorobiphenyl (PCB 132)
to Hydroxylated Metabolites by Human Liver Microsomes and Its Implications
for PCB 132 Neurotoxicity. Toxicol. Sci..

[137] Uwimana E., Ruiz P., Li X., Lehmler H. J. (2019). Human CYP2A6,
CYP2B6, AND CYP2E1 Atropselectively Metabolize Polychlorinated Biphenyls
to Hydroxylated Metabolites. Environ. Sci. Technol..

[138] Li X., Liu Y., Martin J. W., Cui J. Y., Lehmler H.-J. (2021). Nontarget
analysis reveals gut microbiome-dependent differences in the fecal
PCB metabolite profiles of germ-free and conventional mice. Environ. Pollut..

[139] Li X., Lim J. J., Rong C., Lehmler H. J., Cui J. Y. (2025). Deconjugation
of Polychlorinated Biphenyl Sulfates to Hydroxylated PCBs by Anaerobically
Cultured Mouse and Human Gut Microbiota. Chem.
Res. Toxicol..

[140] Rakateli L., Huchzermeier R., van der Vorst E. P. C. (2023). AhR,
PXR and CAR: From Xenobiotic Receptors to Metabolic Sensors. Cells.

[141] Pěnčíková K., Svržková L., Strapáčová S., Neča J., Bartoňková I., Dvořák Z., Hýžd’alová M., Pivnička J., Pálková L., Lehmler H. J. (2018). In
vitro profiling of toxic effects of prominent environmental lower-chlorinated
PCB congeners linked with endocrine disruption and tumor promotion. Environ. Pollut..

[142] Dhakal K., Uwimana E., Adamcakova-Dodd A., Thorne P. S., Lehmler H.-J., Robertson L. W. (2014). Disposition
of Phenolic and Sulfated Metabolites after Inhalation Exposure to
4-Chlorobiphenyl (PCB3) in Female Rats. Chem.
Res. Toxicol..

[143] Jensen M. S., Nørgaard-Pedersen B., Toft G., Hougaard D. M., Bonde J. P., Cohen A., Thulstrup A. M., Ivell R., Anand-Ivell R., Lindh C. H., Jönsson B. A. (2012). Phthalates
and Perfluorooctanesulfonic Acid in Human Amniotic Fluid: Temporal
Trends and Timing of Amniocentesis in Pregnancy. Environ. Health Perspect..

[144] Bräuner E. V., Uldbjerg C. S., Lim Y.-H., Gregersen L. S., Krause M., Frederiksen H., Andersson A.-M. (2022). Presence
of parabens, phenols and phthalates in paired maternal serum, urine
and amniotic fluid. Environ. Int..

[145] Stein C. R., Wolff M. S., Calafat A. M., Kato K., Engel S. M. (2012). Comparison of polyfluoroalkyl compound
concentrations
in maternal serum and amniotic fluid: A pilot study. Reprod. Toxicol..

[146] Fenton S. E., Reiner J. L., Nakayama S. F., Delinsky A. D., Stanko J. P., Hines E. P., White S. S., Lindstrom A. B., Strynar M. J., Petropoulou S.-S. E. (2009). Analysis
of PFOA in dosed CD-1 mice.
Part 2: Disposition of PFOA in tissues and fluids from pregnant and
lactating mice and their pups. Reprod. Toxicol..

[147] Loccisano A. E., Campbell J. L., Butenhoff J. L., Andersen M. E., Clewell H. J. (2012). Evaluation of
placental and lactational pharmacokinetics of PFOA and PFOS in the
pregnant, lactating, fetal and neonatal rat using a physiologically
based pharmacokinetic model. Reprod. Toxicol..

[148] World Health Organization . Safety evaluation of certain food additives and contaminants, supplement 1: non-dioxin-like polychlorinated biphenyls, prepared by the eightieth meeting of the Joint FAO/WHO Expert Committee on Food Additives (JECFA). 2016.

[149] WHO . Environmental Health Criteria 140: Polychlorinated Biphenyls and Terphenyls; World Health Organization: Geneva, 1993.

[150] Lehmann G.
M., Christensen K., Maddaloni M., Phillips L. J. (2015). Evaluating health risks from inhaled
polychlorinated
biphenyls: research needs for addressing uncertainty. Environ. Health Perspect..

[151] Xie X.-L., Zhou W.-T., Zhang K.-K., Yuan Y., Qiu E.-M., Shen Y.-W., Wang Q. (2019). PCB52 induces
hepatotoxicity
in male offspring through aggravating loss of clearance capacity and
activating the apoptosis: Sex-biased effects on rats. Chemosphere.

[152] Xu L. L., Zhang Q. Y., Chen Y. K., Chen L. J., Zhang K. K., Wang Q., Xie X. L. (2022). Gestational
PCB52
exposure induces hepatotoxicity and intestinal injury by activating
inflammation in dam and offspring mice: A maternal and progeny study. Environ. Pollut..

