# Supplementary material for: Maternal–Fetal Transfer and Toxicokinetics of 2,2′,5,5′-Tetrachlorobiphenyl, [14C]-PCB52, Following Intratracheal Administration
Source: Chem Res Toxicol. 2025 Nov 3;38(11):1944–60. doi: 10.1021/acs.chemrestox.5c00265 (PMC12794188; doi:10.1021/acs.chemrestox.5c00265)
Supplement: Supplementary file 1 [file tx5c00265_si_001.pdf]

Supplementary Materials:

Maternal–fetal transfer and toxicokinetics of 2,2',5,5'-tetrachloro biphenyl, [^{14}C]-PCB52, following intratracheal administration

Yau Adamu[1], Andrea Adamcakova-Dodd[2], Xuefang Jing[2], Dustin May[3], and Peter S Thorne[1,2]*

[1]Human Toxicology Program, [2]Department of Occupational and Environmental Health, [3]State Hygienic Laboratory, The University of Iowa, Iowa City, IA, 52242, USA.

*Corresponding author:

Peter S. Thorne, MS, PhD

University of Iowa Distinguished Chair

Department of Occupational and Environmental

145 N. Riverside Dr., 100 CPHB

Iowa City, Iowa 52242, USA

ORCID: 0000-0002-5045-0929

Table of Contents

Table S1	Percent Tissue Distribution of [^{14}C]-PCB52 over time following lung exposure	page 3
Table S2	Toxicokinetic parameters derived from total [^{14}C]-PCB52 concentration-time data	page 5
Figure S1	Schematic illustration of air and excreta sampling during the post-exposure observatory period.	page 6
Figure S2	Pearson correlation coefficients of [^{14}C]-PCB52 concentration in maternal tissues, digestive matter, developing fetus, and amniotic fluids following intratracheal dosing at early pregnancy (GD 11±1. The colors indicate the degree of correlation (r), and the blank squares signify no significant coefficient at a statistical significance level = 0.05.	page 6

Table S1: Percent Tissue Distribution of [^{14}C]-PCB52 over time following lung exposure.

Tissue compartments	0.21 h post-exposure		1.67 h post-exposure		12 h post-exposure		24 h post-exposure		96 h post-exposure	
	Distribution	Conc., dpm/mg	Distribution	Conc., dpm/mg	Distribution	Conc., dpm/mg	Distribution	Conc., dpm/mg	Distribution	Conc., dpm/mg
Muscle	28.65%	1.58	16.65%	12.39	5.23%	2.86	10.09%	6.76	15.01%	1.58
Liver	17.36%	55.77	6.58%	23.78	1.88%	11.09	1.63%	8.22	0.41%	1.16
Adipose tissue	10.92%	8.78	33.81%	19.77	26.32%	27.29	28.25%	33.52	12.91%	11.83
Skin	9.66%	14.76	18.89%	29.78	24.12%	12.17	19.52%	13.13	8.73%	3.58
Adrenal gland	3.76%	3.40	0.08%	0.35	0.04%	10.33	0.02%	5.68	0.02%	3.40
Lung	3.48%	6.22	1.40%	9.65	0.71%	6.35	0.58%	6.31	0.13%	1.70
Serum	3.05%	5.04	1.36%	7.66	0.69%	9.41	0.92%	8.23	0.21%	2.68
Kidney	2.47%	40.98	0.63%	11.01	0.30%	2.65	0.38%	2.44	0.08%	0.53
Small intestine	1.17%	11.75	3.31%	2.40	1.58%	0.85	1.25%	1.66	0.47%	0.56
Heart	0.95%	24.66	0.32%	5.89	0.10%	3.15	0.08%	3.56	0.01%	0.61
Pancreas	0.91%	14.81	0.39%	4.88	0.07%	1.83	0.30%	5.17	0.14%	38.84
Trachea	0.83%	142.06	0.24%	40.98	0.08%	13.20	0.04%	6.29	0.01%	1.03
Brain	0.79%	1.44	0.52%	4.90	0.08%	0.67	0.07%	0.64	0.10%	0.67
Duodenum	0.71%	21.37	0.25%	8.50	0.12%	3.28	0.17%	4.69	0.05%	0.83
Stomach	0.71%	36.55	0.23%	17.01	0.33%	4.80	0.44%	6.56	0.10%	2.28
Matter in intestine	0.70%	10.76	4.97%	1.07	1.53%	0.75	2.58%	13.86	0.02%	0.41
Tongue	0.67%	0.16	0.0003%	4.42	0.06%	1.08	0.05%	1.03	0.01%	0.16
Bronchi	0.66%	81.91	0.20%	33.51	0.10%	20.05	0.08%	18.87	0.01%	1.44
Esophagus	0.51%	72.35	0.06%	5.67	0.01%	1.09	0.01%	0.76	0.003%	0.25
Matter in stomach	0.47%	28.20	0.06%	5.71	0.09%	1.71	1.52%	1.57	0.04%	0.18
Matter in duodenum	0.44%	6.81	0.57%	51.01	0.16%	14.38	0.19%	16.72	0.33%	0.68
Salivary gland	0.39%	8.48	0.18%	2.93	0.04%	1.44	0.06%	2.06	0.04%	0.34
Colon	0.35%	9.24	0.28%	5.24	0.41%	5.56	0.51%	9.18	0.16%	1.87
Spleen	0.29%	13.12	0.12%	3.05	0.04%	2.62	0.07%	5.75	0.03%	0.87
Mammary glands	0.28%	5.39	1.21%	2.46	1.18%	0.74	0.90%	1.55	0.18%	1.67
Uterus	0.21%	0.37	0.11%	2.43	0.06%	0.93	0.16%	2.20	0.13%	0.37
Brown adipose	0.21%	40.87	0.87%	120.53	0.00%	27.78	0.18%	23.09	0.00%	4.17
Ovary	0.20%	19.85	0.08%	7.08	0.02%	1.61	0.05%	5.24	0.02%	1.95
Rectal amp.	0.20%	14.88	0.12%	5.18	0.30%	1.10	0.22%	1.73	0.09%	1.12
Placenta	0.17%	9.19	0.21%	6.13	0.04%	9.49	0.11%	10.39	0.02%	4.93

Matter in colon	0.14%	2.42	0.11%	1.23	4.20%	65.87	5.74%	66.81	0.36%	10.10
Matter in amp. colon	0.12%	2.42	0.08%	0.81	2.61%	32.64	1.82%	27.35	0.30%	3.55
Thymus	0.11%	9.77	0.11%	5.38	0.03%	1.76	0.03%	1.62	0.01%	0.83
Fetus	0.07%	0.06	0.08%	2.02	0.002%	0.47	0.03%	0.19	0.02%	0.06
Lymph nodes	0.02%	9.72	0.05%	21.16	0.04%	23.12	0.03%	26.43	0.02%	4.53
Bladder	0.01%	0.00	0.05%	2.82	0.01%	1.20	0.02%	3.71	ND	0.00
Pituitary	0.01%	0.08	0.002%	2.59	0.001%	1.15	0.002%	1.15	0.02%	0.08
Amniotic fluid	0.0024%	0.01	0.018%	10.77	0.0008%	0.06	0.0009%	0.12	0.0004%	0.01
Thyroid	ND	ND	0.019%	16.43	0.003%	3.25	0.004%	3.26	0.001%	0.66

ND; not detected, Conc.; Concentration of ^{14}C-PCB52 per mg of wet tissue

Table S2. Toxicokinetic parameters derived from total [^{14}C]-PCB52 concentration-time data.

Tissues	Ke {h^{-1}}	λ_z {h}	T_{max} {h}	C_{max} {dpm/mg}	C_{96} {dpm/mg}	AUC_{0-96} {h*dpm/mg}	$AUC_{0-\infty}$ {h*dpm/mg}	Pt ($AUC_{0-\infty tissue}$ / $AUC_{0-\infty blood}$	AUC_%Extrap
Lung	0.03	22.05	0.20	770.74	14.01	9124.64	9570.22	8.41	4.66
Heart	0.03	26.21	0.20	265.34	1.80	1026.89	1094.86	0.96	6.21
Kidney	0.02	35.41	0.20	232.02	5.98	2028.88	2334.38	2.05	13.09
Liver	0.02	42.13	0.20	385.58	5.19	1880.91	2196.67	1.93	14.37
Muscle	0.00	1231.42	0.20	50.72	16.28	992.53	29911.99	26.28	96.68
Skin	0.01	58.90	12.00	91.59	26.05	4962.64	7176.24	6.31	30.85
Adipose tissue	0.01	72.85	12.00	265.66	115.14	16495.13	28597.12	25.13	42.32
Ovary	0.01	46.53	1.67	120.59	15.41	2954.80	3989.28	3.51	25.93
Uterus	0.02	34.85	1.67	23.62	3.61	862.31	1043.55	0.92	17.37
Adrenal gland	0.00	253.59	12.00	100.56	33.10	4167.45	16276.54	14.30	74.40
Bladder	0.01	57.81	1.67	104.87	9.20	1126.61	1893.58	1.66	40.50
Thymus	0.01	53.40	0.20	91.92	8.05	1847.60	2468.08	2.17	25.14
Thyroid	0.02	37.74	1.67	159.95	6.46	2364.57	2716.31	2.39	12.95
Spleen	0.01	55.79	0.20	110.57	5.48	816.80	1257.75	1.11	35.06
Matter in amp colon	0.02	30.46	12.00	317.72	34.55	10077.65	11595.67	10.19	13.09
Matter in Colon	0.02	40.00	12.00	641.30	98.31	18672.10	24345.51	21.39	23.30
Matter in intestine	0.04	17.41	1.67	496.59	6.62	7470.99	7637.39	6.71	2.18
Matter in stomach	0.04	16.50	0.20	101.24	4.02	2254.70	2350.33	2.07	4.07
Matter in duodenum	0.02	34.63	0.20	343.87	22.17	3047.63	4155.46	3.65	26.66
Colon tissue	0.02	40.46	0.20	86.99	18.20	3729.32	4791.52	4.21	22.17
Duodenum	0.02	34.84	0.20	201.05	8.12	2486.20	2894.43	2.54	14.10
Esophagus	0.01	46.31	0.20	680.81	2.43	1222.83	1384.98	1.22	11.71
Pancreas	0.01	67.74	0.20	186.81	18.96	3119.59	4972.41	4.37	37.26
Rectal Amp.	0.01	133.61	12.00	92.37	48.04	5633.45	14892.47	13.08	62.17
Salivary gland	0.01	84.60	0.20	139.99	10.94	1692.89	3028.07	2.66	44.09
Small Intestine	0.01	47.33	1.67	289.95	34.90	6903.90	9286.69	8.16	25.66
Stomach	0.02	32.02	0.20	123.47	8.52	2224.27	2617.74	2.30	15.03
Fetus	0.02	34.78	1.67	19.63	0.57	280.01	308.49	0.27	9.23
Placenta	0.03	21.11	1.67	25.24	0.74	520.08	542.56	0.48	4.14
Amniotic Fluid	0.03	26.38	1.67	3.40	0.12	51.81	56.38	0.05	8.11
Mammary glands	0.02	34.16	12.00	225.07	44.06	12158.48	14329.69	12.59	15.15
Pituitary gland	ND	ND	96.00	378.12	378.12	10961.38	ND	ND	ND

NA, not applicable; ND, not determinable; $AUC_{0-\infty}$, area under the concentration-time curve from zero up to infinity, based on the terminal phase calculated with the equation $AUC_{0-\infty} = AUC_{0-24} + C_{last}/Ke$ using known observed concentration & terminal elimination half-life values. Cl/F, apparent total serum clearance of 14C-PCB52 after intratracheal dose estimated from CL/F =Dose/$AUC_{0-\infty}$; Vz/F, apparent volume of distribution during terminal phase for non-steady-state after extravascular administration estimated from Vz/F = $Dose_{\_ex}/(AUC_{0-\infty}*Ke)$; Pt, partition coefficients estimated based on the ratios of AUC from zero to infinity in blood to the AUC in the respected tissues after pulmonary exposure to high dose and low dose of [^{14}C]-PCB52 (mean, n = 2–3).

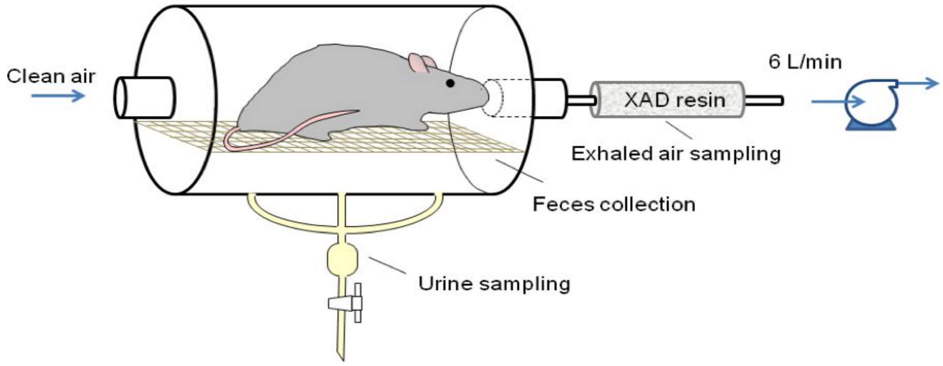

Figure S1 Schematic illustration of air and excreta sampling during the post-exposure observatory period.

Source: Hu, X., Adamcakova-Dodd, A., & Thorne, P. S. (2014). The fate of inhaled ^{14}C-labeled PCB11 and its metabolites in vivo. *Environment International, 63*, 92-100

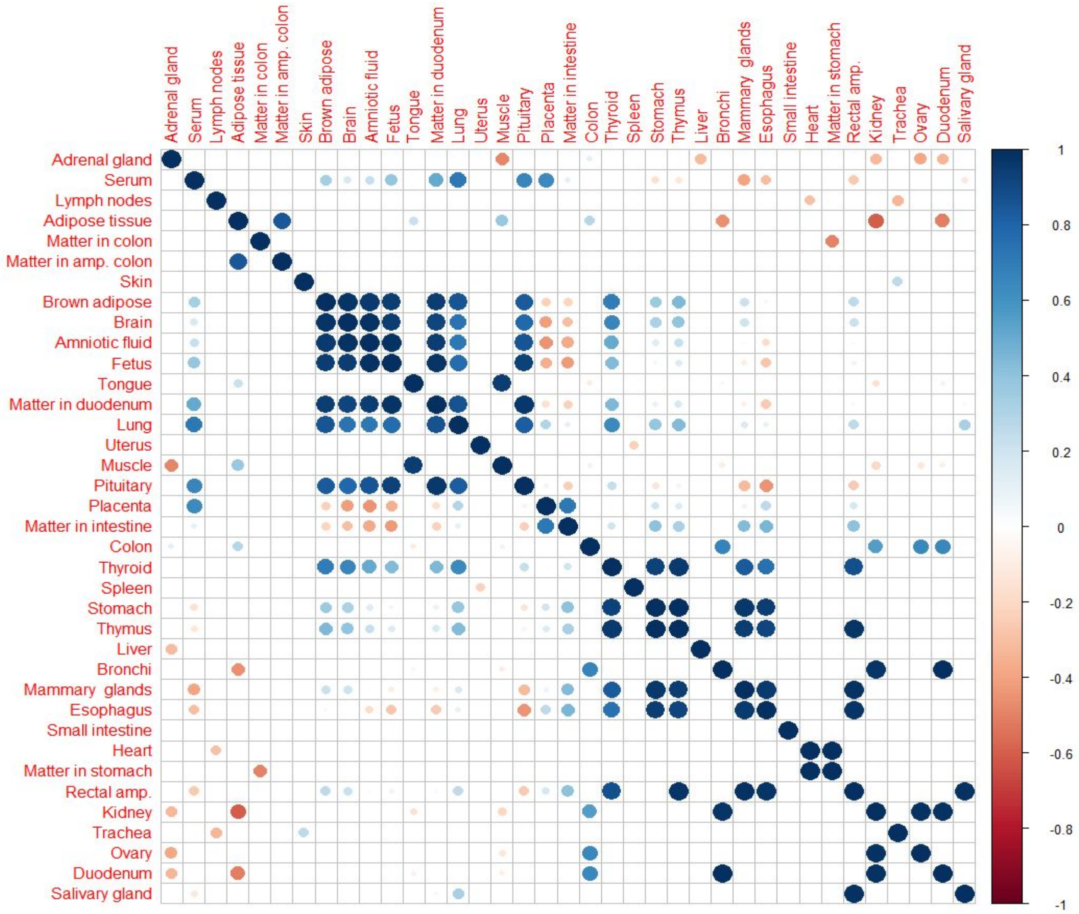

Figure S2 Pearson correlation coefficients of [^{14}C]-PCB52 concentration in maternal tissues, digestive matter, developing fetus, and amniotic fluids following intratracheal dosing at early pregnancy (GD 11±1. The colors indicate the degree of correlation (r), and the blank squares signify no significant coefficient at a statistical significance level = 0.05.
